# Comparative predictive value of nine inflammation-derived haematological indices for 28-day mortality in patients with sepsis: a multicentre retrospective cohort study

**DOI:** 10.3389/fmed.2026.1857973

**Published:** 2026-06-19

**Authors:** Kaihuan Zhou, Chen Ou, Xiaorong Li, Zhanhong Tang, Juntao Hu

**Affiliations:** Department of Critical Care Medicine, The First Affiliated Hospital of Guangxi Medical University, Nanning, China

**Keywords:** 28-day mortality, external validation, inflammation-derived haematological indices, risk stratification, sepsis

## Abstract

**Background:**

Sepsis is associated with high mortality, and early risk stratification is essential. Inflammation-derived hematological indices from routine complete blood count testing are readily available, inexpensive, and reproducible, but their comparative prognostic value in sepsis remains unclear.

**Methods:**

This multicenter retrospective cohort study used the MIMIC-IV database as the development cohort and the First Affiliated Hospital of Guangxi Medical University as the external validation cohort. Adult patients meeting Sepsis-3 criteria with an intensive care unit length of stay >24 h were included. Nine inflammation-derived hematological indices were evaluated: neutrophil-to-lymphocyte ratio, platelet-to-lymphocyte ratio, monocyte-to-lymphocyte ratio, neutrophil-to-monocyte ratio, neutrophil-to-platelet ratio, monocyte-to-platelet ratio, systemic immune-inflammation index, systemic inflammation response index, and aggregate index of systemic inflammation. Cox regression, restricted cubic spline analysis, receiver operating characteristic curves, calibration curves, decision curve analysis, and Sequential Organ Failure Assessment combined analyses assessed their prognostic value for 28-day all-cause mortality after the 24 h landmark time.

**Results:**

The development cohort included 26,512 patients with sepsis, among whom 2,669 died within 28 days. All nine indices were significantly associated with 28-day mortality after the landmark time. In the fully adjusted model, monocyte-to-lymphocyte ratio, neutrophil-to-platelet ratio, neutrophil-to-lymphocyte ratio, and systemic inflammation response index showed relatively larger effect estimates. Unadjusted discrimination was generally moderate, with the neutrophil-to-lymphocyte ratio showing the highest area under the curve. Restricted cubic spline analysis showed non-linear associations between all indices and mortality risk, although inflection points should not be interpreted as clinical thresholds. Fully adjusted and Sequential Organ Failure Assessment combined analyses suggested supplementary prognostic information, particularly from neutrophil-to-lymphocyte ratio, systemic inflammation response index, and monocyte-to-lymphocyte ratio. The external validation cohort included 850 patients, among whom 182 died within 28 days. After full adjustment, none of the indices remained significantly associated with 28-day mortality.

**Conclusion:**

Inflammation-derived hematological indices were associated with 28-day mortality and showed non-linear risk patterns in the development cohort. However, their standalone discriminatory ability was limited, and externally validated associations were not consistent. These indices may serve as supplementary markers alongside conventional severity scores rather than independent mortality prediction tools. Further prospective multicentre validation is warranted.

## 1 Background

Sepsis is defined as life-threatening organ dysfunction caused by a dysregulated host response to infection (1). Epidemiological studies have estimated that approximately 50 million cases of sepsis occur worldwide each year, with a very high risk of death, making sepsis a major global public health challenge (2, 3). Although advances in comprehensive management, including antimicrobial therapy and organ support, have improved outcomes to some extent, mortality remains unacceptably high, particularly among patients with complex illness and multiple organ dysfunction (4). In this context, early identification of high-risk patients and timely risk-stratified management are essential for reducing mortality and improving prognosis. Therefore, the development of simple, objective, and reliable prognostic tools is of considerable importance for clinical decision-making and resource allocation.

Several prognostic scoring systems, including the Acute Physiology and Chronic Health Evaluation II (APACHE II), Simplified Acute Physiology Score II (SAPS II), and Sequential Organ Failure Assessment (SOFA), are widely used in sepsis and play important roles in disease severity stratification and outcome assessment (5–7). However, these scoring systems rely on multiple clinical and laboratory variables, and their calculation is relatively complex, which limits their utility in real-time monitoring and rapid decision-making (8). In addition, differences in data availability and implementation processes across healthcare settings may further restrict their generalizability across diverse populations and clinical scenarios. There is therefore a clear need to identify simpler and more readily available biological markers to complement and optimize current prognostic assessment strategies.

In recent years, inflammation-derived indices based on routine complete blood count parameters, such as the neutrophil-to-lymphocyte ratio (NLR) and platelet-to-lymphocyte ratio (PLR), have attracted increasing attention because of their accessibility, low cost, and high reproducibility (9, 10). By quantifying the dynamic balance among different immune cell subsets, these indices may provide an integrated reflection of inflammatory activation, immune suppression, and physiological stress. Previous studies have suggested that some of these indices are associated with sepsis outcomes. However, existing evidence has largely focused on individual markers, and systematic parallel comparisons of multiple inflammation-derived indices within the same large cohort remain scarce (9, 11).

Accordingly, using the Medical Information Mart for Intensive Care IV (MIMIC-IV) database together with an external validation cohort, we conducted a multicenter retrospective cohort study to systematically compare the prognostic value of nine inflammation-derived hematological indices in a large population of patients with sepsis. We assessed not only their independent associations with 28-day mortality, but also comprehensively compared their patterns of risk association, predictive performance, and potential clinical applicability using multivariable regression, restricted cubic spline analysis, receiver operating characteristic analysis, calibration curves, decision curve analysis, and subgroup analyses. External validation was further performed to examine the robustness and generalizability of the findings. This study aimed to provide evidence to support the application of inflammation-derived hematological indices in early risk stratification and precise prognostic assessment in sepsis.

## 2 Methods

### 2.1 Study design and data source

This was a multicenter retrospective cohort study. The development cohort was derived from the MIMIC-IV database (12), which contains detailed clinical information on hospitalized patients admitted to Beth Israel Deaconess Medical Center between 2008 and 2022. Before data access, the research team completed the required training and certification program (Record ID: 62695673). As all MIMIC-IV data had been de-identified, additional informed consent and ethical approval were waived. An independent external validation cohort from the ICU of the First Affiliated Hospital of Guangxi Medical University was also included, and the study was approved by its institutional ethics committee.

This study used ICU admission as the starting point of the clinical timeline, and 24 h after ICU admission was defined as the landmark time and used as time zero for the primary survival analysis. To ensure a clear temporal sequence among sepsis identification, exposure assessment, and outcome follow-up, we included only adult patients with sepsis who met the Sepsis-3 criteria at ICU admission or within 24 h after ICU admission. All inflammation-derived hematological indices and relevant covariates were derived from clinical data obtained within the first 24 h after ICU admission, whereas mortality follow-up began after the landmark time.

**Inclusion criteria:** Patients were eligible if they (1) met the diagnostic criteria for sepsis according to the Sepsis-3.0 definition (1), (2) were aged ≥18 years, (3) were admitted to the ICU for more than 24 h, (4) had a complete blood count with differential available. For patients with multiple ICU admissions, only the first ICU stay was included for analysis.

**Exclusion criteria:** Patients were excluded if they had (1) missing key laboratory data required to construct the main exposure variables, including absolute neutrophil count, absolute lymphocyte count, absolute monocyte count, or platelet count; (2) severe hepatic dysfunction; (3) end-stage renal disease; (4) a diagnosis of malignancy or immunodeficiency; or (5) no record of ICU admission.

Patients with severe liver failure, end-stage renal disease, malignancy, or immunodeficiency-related diagnoses were excluded mainly because these populations have distinct complete blood count profiles and immune cell distributions. Their peripheral blood cell counts may be affected by the underlying disease, treatment-related factors, and chronic organ failure, and may therefore not purely reflect the inflammatory response related to sepsis. Accordingly, these patients were excluded to reduce the influence of highly heterogeneous underlying diseases on the interpretation of the nine inflammation-derived hematological indices.

In this study, 24 h after ICU admission was defined as the landmark time and used as time zero for survival analysis. The primary outcome was 28-day all-cause mortality calculated from the landmark time.

### 2.2 Data extraction and variables

Data for patients who met the inclusion and exclusion criteria were extracted from the MIMIC-IV (V 3.1) database and categorized into four major domains:

(1) Demographic and general information: age, sex, ethnicity, systolic blood pressure (SBP), diastolic blood pressure (DBP), mean arterial pressure (MAP), and respiratory rate (RR). (2) Comorbidities: hypertension (HTN), acute kidney injury (AKI), pneumonia (PNA), cerebrovascular accident (CVA), chronic kidney disease (CKD), type 1 diabetes mellitus (T1DM), type 2 diabetes mellitus (T2DM), hyperlipidemia (HLD), heart failure (HF), myocardial infarction (MI), ischemic heart disease (IHD), and chronic obstructive pulmonary disease (COPD). (3) Laboratory parameters within the first 24 h after ICU admission: Complete blood count and biochemical indices, including absolute lymphocyte count (ALC), absolute neutrophil count (ANC), absolute monocyte count (AMC), red blood cell count (RBC), hemoglobin (Hb), hematocrit (HCT), red cell distribution width (RDW), platelet count (PLT), white blood cell count (WBC), serum albumin, creatinine, anion gap (AG), pH, lactate (Lac), and partial thromboplastin time (PTT). (4) Disease severity scores: SOFA, Acute Physiology Score III (APSIII), Simplified Acute Physiology Score II (SAPSII), Charlson Comorbidity Index (CCI), and Acute Physiology and Chronic Health Evaluation II (APACHE II).

In addition, nine inflammation-derived hematologic indices were calculated based on blood parameters: Neutrophil-to-lymphocyte ratio (NLR = ANC / ALC); Platelet-to-lymphocyte ratio (PLR = PLT / ALC); Monocyte-to-lymphocyte ratio (MLR = AMC / ALC); Neutrophil-to-monocyte ratio (NM = ANC / AMC); Neutrophil-to-platelet ratio (NP = ANC / PLT); Monocyte-to-platelet ratio (MP = AMC / PLT); Systemic immune-inflammation index (SII = PLT × ANC / ALC); Systemic inflammation response index (SIRI = ANC × AMC / ALC); Aggregate index of systemic inflammation (AISI = ANC × AMC × PLT / ALC).

### 2.3 Missing data imputation and outlier management strategies

The proportion of missing data was first assessed for all candidate variables. With reference to commonly used strategies in previous studies based on large ICU databases, a missingness threshold of 20% was set for variable inclusion (13, 14). This threshold was intended to balance the retention of variable information against the reliability of imputation. Variables with ≤20% missingness were handled using multiple imputation with chained equations (MICE), generating five imputed datasets, and the results were pooled according to Rubin’s rules. Predictive mean matching was used for continuous variables, and binary logistic regression was used for binary variables.

For outlier management, we considered that ICU patients often have complex clinical conditions and substantial heterogeneity in disease severity, and that some laboratory variables and vital signs may show markedly skewed distributions and extreme values. These extreme values may reflect true critical pathophysiological states, but they may also be influenced by measurement error, recording error or abnormal database entry. Directly deleting extreme values could lead to the loss of important clinical information, whereas retaining all extreme observations without adjustment could exert a disproportionate influence on regression coefficient estimation, model training, and predictive performance evaluation. Therefore, winsorization at the 1st and 99th percentiles was applied in this study. Values of continuous variables below the 1st percentile or above the 99th percentile were capped at the corresponding percentile values. This approach preserves the sample size and the main features of the variable distributions as far as possible, while reducing the undue influence of extreme observations on statistical inference and model development, thereby improving the robustness of the analyses.

### 2.4 Statistical analysis

#### 2.4.1 Comparison of baseline characteristics

Descriptive statistical analyses were performed to compare baseline clinical and laboratory characteristics between outcome groups. Categorical variables were compared using the chi-square test or Fisher’s exact test and are presented as counts and percentages. Continuous variables were compared using the Wilcoxon rank sum test, Student’s t test, or one-way analysis of variance (ANOVA), as appropriate, and are presented as mean ± standard deviation (SD).

#### 2.4.2 Cox regression analysis

To evaluate the associations between the nine inflammation-derived hematological indices and the risk of 28-day mortality after ICU admission in patients with sepsis, Cox proportional hazards regression models were constructed separately for each index. All nine indices were standardized per 1 SD increase to facilitate direct comparison of effect sizes across indices. For each index, three hierarchical models were fitted sequentially. Model 1 was unadjusted. Model 2 was adjusted for demographic variables, including age, sex, and race. Model 3 was further adjusted for comorbidities and laboratory variables on the basis of Model 2, including AKI, CKD, COPD, HTN, IHD, MI, HF, PNA, T2DM, albumin, AG, creatinine, blood urea nitrogen, total bilirubin, glucose, sodium, potassium, chloride, total calcium, international normalized ratio, aspartate aminotransferase, and alanine aminotransferase. Results are reported as hazard ratios (HRs), 95% confidence intervals (CIs), and *p* values.

#### 2.4.3 Restricted cubic spline modeling

Restricted cubic spline (RCS) analysis was used to explore potential non-linear associations between the inflammation-derived indices and the risk of mortality. This approach enabled visualization of the dose–response relationships between different levels of each inflammation index and mortality risk, and allowed formal testing of non-linearity using the *p* value for nonlinearity.

#### 2.4.4 Discrimination, calibration, and clinical net benefit

To assess the discriminative ability of the nine inflammation-derived hematological indices for predicting 28-day mortality, receiver operating characteristic (ROC) curves were constructed for the unadjusted model, the demographically adjusted model, and the fully adjusted model, and the area under the curve (AUC) was calculated. Calibration curves were further used to evaluate the agreement between predicted probabilities and observed risk. Decision curve analysis (DCA) was performed to assess the net clinical benefit across a range of threshold probabilities.

#### 2.4.5 Incremental predictive value beyond the SOFA score

To evaluate the incremental predictive value of the nine inflammation-derived hematological indices beyond the SOFA score, two sets of nested Cox regression models were constructed. The baseline model included only the SOFA score, whereas each combined model incorporated the SOFA score plus one inflammation-derived hematological index.

It should be noted that this study did not develop a new additive composite score, nor were the inflammatory indices categorized and incorporated into the SOFA score using fixed weights. All inflammation-derived hematological indices were included in the models as continuous variables, to preserve their original information as far as possible and avoid information loss caused by categorization.

The AUC of the SOFA-only model was then compared with that of each SOFA combined model. The incremental predictive value of each inflammation-derived hematological index beyond SOFA was further assessed using the change in AUC (ΔAUC), likelihood ratio tests for nested models, the integrated discrimination improvement index, and the continuous net reclassification improvement index.

#### 2.4.6 Sensitivity analyses

To assess the robustness of the main Cox regression results, two sensitivity analyses were performed. First, the fully adjusted Cox regression results from the multiply imputed dataset were compared with those from the complete-case dataset to evaluate the influence of the missing data handling strategy on the main associations. Second, the fully adjusted Cox regression results before winsorization were compared with those after winsorization at the 1st and 99th percentiles to assess the impact of outlier handling on effect estimates and statistical conclusions.

### 2.5 External validation

To assess the stability of the nine inflammation-derived hematological indices across different populations, patients admitted to the ICU of the First Affiliated Hospital of Guangxi Medical University were included as an independent external validation cohort. The external validation cohort used the same inclusion and exclusion criteria, outcome definition, variable extraction strategy, and calculation methods for the nine inflammation-derived hematological indices as those used in the development cohort. For variables with differences in nomenclature or units of measurement, harmonization and standardization were performed before analysis to ensure comparability between the two cohorts.

Baseline characteristics were compared between 28-day survivors and non-survivors in the external validation cohort. Cox proportional hazards regression models were then constructed to evaluate the associations between each of the nine inflammation-derived hematological indices and 28-day mortality. Consistent with the analytical strategy used in the development cohort, unadjusted, demographically adjusted, and fully adjusted models were fitted, and all indices were analyzed per 1 SD increase. Results are reported as HRs, 95% CIs, and *p* values.

All statistical analyses were performed using R software, version 4.4.1. All tests were two-sided, and *p* < 0.05 was considered statistically significant.

## 3 Results

### 3.1 Baseline characteristics of the study population

**Figure 1:**
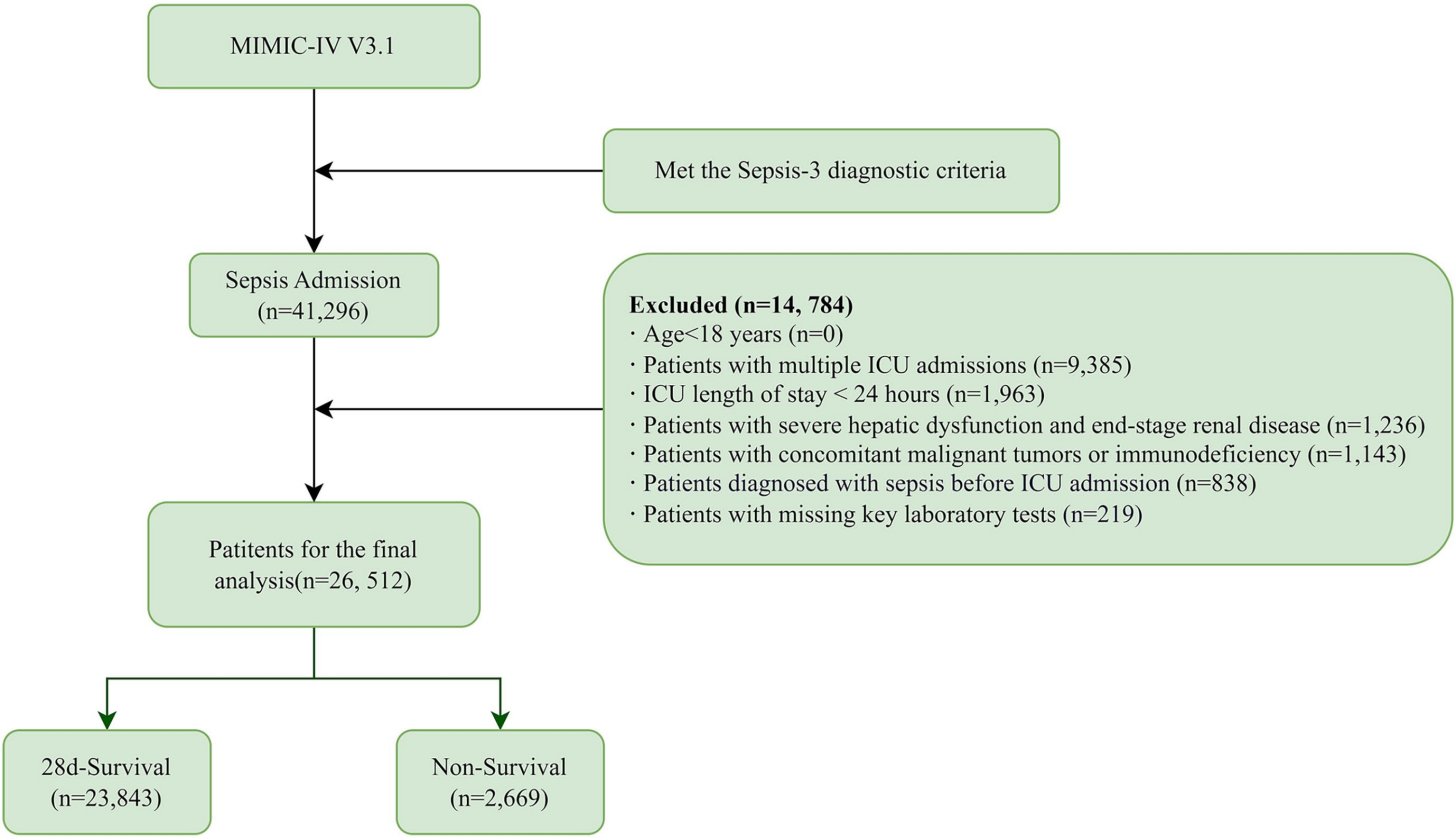
Flowchart of patient selection.

According to the inclusion and exclusion criteria (Figure 1), the development cohort finally included 26,512 patients with sepsis, of whom 2,669 died within 28 days after intensive care unit (ICU) admission and 23,843 survived (Table 1). Compared with survivors, non-survivors were older, had a higher respiratory rate, and had a greater burden of comorbidities, including higher proportions of acute kidney injury, pneumonia, chronic kidney disease, type 2 diabetes mellitus, heart failure, myocardial infarction, ischemic heart disease, and chronic obstructive pulmonary disease. Laboratory findings indicated more pronounced inflammatory activation, immune dysregulation, and organ dysfunction in non-survivors, as reflected by higher levels of neutrophils, monocytes, white blood cells, red cell distribution width (RDW), anion gap, creatinine, and lactate, and lower levels of lymphocytes, platelets, red blood cells, hemoglobin, hematocrit, albumin, and pH. The SOFA, APS III, SAPS II, and APACHE II scores were significantly higher in non-survivors, indicating greater disease severity. All nine inflammation-derived hematological indices were significantly elevated in non-survivors, with relatively larger standardized differences for the SIRI, NLR, MLR, AISI, and SII, suggesting their potential value for early risk stratification.

**Table 1:** Baseline characteristics of patients according to 28-day mortality status.

Variable	Overall (*N* = 26,512)	Survivor (*N* = 23,843)	No-survivor (*N* = 2,669)	*p*-value	SMD
Characteristics					
Age	63.59 ± 17.26	62.70 ± 17.29	71.55 ± 14.80	<0.001	0.550
MAP	81.42 ± 15.62	81.40 ± 15.55	81.62 ± 16.22	0.518	0.013
RR	19.88 ± 5.75	19.79 ± 5.73	20.70 ± 5.89	<0.001	0.157
Gender				0.670	0.009
F	11,921 (44.96%)	10,710 (44.92%)	1,211 (45.37%)		
M	14,591 (55.04%)	13,133 (55.08%)	1,458 (54.63%)		
Race				<0.001	
Black	2,696 (10.17%)	2,484 (10.42%)	212 (7.94%)		
Other	6,878 (25.94%)	6,011 (25.21%)	867 (32.48%)		
White	16,938 (63.89%)	15,348 (64.37%)	1,590 (59.57%)		
Comorbidities					
HTN, *n* (%)				<0.001	0.084
No	16,489 (62.19%)	14,732 (61.79%)	1757 (65.83%)		
Yes	10,023 (37.81%)	9,111 (38.21%)	912 (34.17%)		
AKI, *n* (%)				<0.001	0.786
No	18,989 (71.62%)	17,946 (75.27%)	1,043 (39.08%)		
Yes	7,523 (28.38%)	5,897 (24.73%)	1,626 (60.92%)		
PNA, *n* (%)				<0.001	0.582
No	21,906 (82.63%)	20,298 (85.13%)	1,608 (60.25%)		
Yes	4,606 (17.37%)	3,545 (14.87%)	1,061 (39.75%)		
CVA, *n* (%)				<0.001	0.066
No	24,907 (93.95%)	22,439 (94.11%)	2,468 (92.47%)		
Yes	1,605 (6.05%)	1,404 (5.89%)	201 (7.53%)		
CKD, *n* (%)				<0.001	0.272
No	22,108 (83.39%)	20,146 (84.49%)	1962 (73.51%)		
Yes	4,404 (16.61%)	3,697 (15.51%)	707 (26.49%)		
T2DM, *n* (%)				<0.001	0.127
No	19,793 (74.66%)	17,936 (75.23%)	1857 (69.58%)		
Yes	6,719 (25.34%)	5,907 (24.77%)	812 (30.42%)		
T1DM, *n* (%)				0.291	0.024
No	26,171 (98.71%)	23,530 (98.69%)	2,641 (98.95%)		
Yes	341 (1.29%)	313 (1.31%)	28 (1.05%)		
HLD, *n* (%)				0.202	0.027
No	15,852 (59.79%)	14,225 (59.66%)	1,627 (60.96%)		
Yes	10,660 (40.21%)	9,618 (40.34%)	1,042 (39.04%)		
HF, *n* (%)				<0.001	0.337
No	21,204 (79.98%)	19,419 (81.45%)	1785 (66.88%)		
Yes	5,308 (20.02%)	4,424 (18.55%)	884 (33.12%)		
MI, *n* (%)				<0.001	0.233
No	24,088 (90.86%)	21,845 (91.62%)	2,243 (84.04%)		
Yes	2,424 (9.14%)	1998 (8.38%)	426 (15.96%)		
IHD, *n* (%)				<0.001	0.132
No	18,597 (70.15%)	16,873 (70.77%)	1724 (64.59%)		
Yes	7,915 (29.85%)	6,970 (29.23%)	945 (35.41%)		
COPD, *n* (%)				<0.001	0.224
No	23,348 (88.07%)	21,189 (88.87%)	2,159 (80.89%)		
Yes	3,164 (11.93%)	2,654 (11.13%)	510 (19.11%)		
Laboratory tests					
Lymphocyte count	1.39 ± 0.76	1.43 ± 0.76	1.06 ± 0.69	<0.001	0.510
Neutrophil count	7.88 ± 4.74	7.61 ± 4.60	10.26 ± 5.29	<0.001	0.534
Monocyte count	0.72 ± 0.41	0.71 ± 0.40	0.84 ± 0.46	<0.001	0.313
PLT	209.87 ± 90.57	211.18 ± 88.90	198.08 ± 103.60	<0.001	0.136
Hematocrit	33.97 ± 6.66	34.17 ± 6.58	32.17 ± 7.08	<0.001	0.293
Hemoglobin	11.06 ± 2.31	11.14 ± 2.29	10.35 ± 2.36	<0.001	0.340
RDW	14.39 ± 1.81	14.28 ± 1.77	15.36 ± 1.89	<0.001	0.590
RBC	3.74 ± 0.79	3.77 ± 0.78	3.47 ± 0.82	<0.001	0.374
WBC	9.72 ± 4.71	9.54 ± 4.61	11.35 ± 5.26	<0.001	0.365
Albumin	3.34 ± 0.64	3.38 ± 0.63	2.95 ± 0.63	<0.001	0.681
Anion gap	13.57 ± 3.38	13.43 ± 3.31	14.80 ± 3.73	<0.001	0.387
PTT	30.57 ± 4.85	30.51 ± 4.77	31.14 ± 5.45	<0.001	0.123
Creatinine	0.96 ± 0.35	0.94 ± 0.34	1.11 ± 0.41	<0.001	0.444
pH	7.36 ± 0.08	7.36 ± 0.08	7.35 ± 0.09	<0.001	0.130
Lac	1.98 ± 0.94	1.96 ± 0.94	2.12 ± 1.01	<0.001	0.169
Inflammation indices					
SIRI	6.37 ± 9.32	5.70 ± 8.19	12.34 ± 14.99	<0.001	0.550
MP	0.00 ± 0.01	0.00 ± 0.00	0.01 ± 0.01	<0.001	0.279
MLR	0.70 ± 0.69	0.65 ± 0.63	1.09 ± 1.01	<0.001	0.521
NP	0.05 ± 0.06	0.04 ± 0.05	0.07 ± 0.11	<0.001	0.346
NM	14.82 ± 18.61	14.56 ± 18.41	17.11 ± 20.20	<0.001	0.132
NLR	8.40 ± 11.26	7.65 ± 10.18	15.05 ± 16.89	<0.001	0.530
AISI	1411.68 ± 2302.97	1275.06 ± 2042.74	2632.19 ± 3708.71	<0.001	0.453
SII	1777.77 ± 2739.22	1636.36 ± 2551.72	3041.01 ± 3820.50	<0.001	0.432
PLR	219.52 ± 314.40	211.03 ± 306.56	295.41 ± 368.82	<0.001	0.249
Scores					
SOFA	6.41 ± 3.42	6.29 ± 3.33	7.45 ± 3.98	<0.001	0.316
APSIII	50.32 ± 20.87	49.44 ± 20.33	58.15 ± 23.79	<0.001	0.394
SAPSII	40.39 ± 13.42	39.86 ± 13.20	45.10 ± 14.40	<0.001	0.380
Charlson	5.23 ± 2.91	5.16 ± 2.88	5.91 ± 3.05	<0.001	0.255
APACHE II	20.05 ± 6.93	19.80 ± 6.82	22.28 ± 7.43	<0.001	0.347

HTN, hypertension; AKI, acute kidney injury; PNA, pneumonia; CVA, cerebrovascular accident; CKD, chronic kidney disease; T2DM, type 2 diabetes mellitus; T1DM, type 1 diabetes mellitus; HLD, hyperlipidemia; HF, heart failure; MI, myocardial infarction; IHD, ischemic heart disease; COPD, chronic obstructive pulmonary disease; RBC, red blood cell; WBC, white blood cell; RDW, red cell distribution width; PLT, platelet count; NLR, neutrophil-to-lymphocyte ratio; PLR, platelet-to-lymphocyte ratio; SII, systemic immune-inflammation index; SIRI, systemic inflammation response index; AISI, aggregate inflammation response index; MLR, monocyte-to-lymphocyte ratio; NP, neutrophil-to-platelet ratio; NM, neutrophil-to-monocyte ratio; MP, monocyte-to-platelet ratio; APSIII, acute physiology score III; SAPSII, simplified acute physiology score II; SOFA, sequential organ failure assessment; APACHEII, acute physiology and chronic health evaluation II; MAP, mean arterial pressure; RR, respiratory rate; Lac, lactate; PTT, partial thromboplastin time; SMD, standardized mean difference.

### 3.2 Cox regression analysis of inflammation-derived hematological ratios and 28-day mortality in sepsis

In the Cox regression analyses, all nine inflammation-derived hematological indices were winsorized at the 1st and 99th percentiles and then standardized. HRs therefore represent the change in mortality risk per 1 standard deviation increase in each index. Before constructing the Cox regression models, we further assessed model assumptions and applicability. Multicollinearity analysis showed that the variance inflation factors (VIFs) for all covariates and for each of the nine separately included inflammation-derived hematological indices were below 5, indicating no substantial harmful collinearity (Supplementary Tables S3). The proportional hazards assumption was assessed using Schoenfeld residuals. Although the global tests for the fully adjusted models reached statistical significance, the index-specific tests for the nine inflammation-derived hematological indices did not indicate violation of the proportional hazards assumption, supporting the validity of the Cox effect estimates for the main exposure variables (Supplementary Table S7).

In the unadjusted models, all nine inflammation-derived hematological indices were significantly associated with the risk of 28-day mortality in patients with sepsis (Table 2). Ranked by effect size, the HRs for MLR, NLR, SIRI, NP, SII, AISI, MP, PLR, and neutrophil-to-monocyte ratio (NM) were 1.534, 1.531, 1.530, 1.489, 1.451, 1.441, 1.353, 1.305, and 1.147, respectively, all of which were statistically significant. These findings suggest that, before covariate adjustment, higher levels of composite inflammatory indices were closely associated with an increased risk of subsequent mortality, with relatively stronger associations observed for MLR, NLR, SIRI, and NP.

**Table 2:** Unadjusted cox regression for nine inflammation-derived haematological indices.

Inflammation index	HR	95% CI	*p* value
SIRI	1.530	1.496–1.565	<0.001***
MLR	1.534	1.497–1.572	<0.001***
AISI	1.441	1.407–1.475	<0.001***
NM	1.147	1.113–1.183	<0.001***
MP	1.353	1.320–1.387	<0.001***
NP	1.489	1.454–1.526	<0.001***
NLR	1.531	1.497–1.565	<0.001***
SII	1.451	1.417–1.486	<0.001***
PLR	1.305	1.271–1.341	<0.001***

All inflammation-derived haematological indices were winsorised at the 1st and 99th percentiles and then standardized; hazard ratios are presented per 1 SD increase. HR, hazard ratio; CI, confidence interval. *p < 0.05, **p < 0.01, ***p < 0.001.

After further adjustment for age, sex, and race, the associations between the nine inflammation-derived hematological indices and 28-day mortality remained stable and statistically significant (Table 3). MLR, NLR, SIRI, and NP continued to show relatively larger effect estimates, with HRs of 1.486, 1.489, 1.480, and 1.480, respectively. SII, AISI, MP, and PLR were also significantly associated with increased mortality risk, with HRs of 1.418, 1.398, 1.346, and 1.291, respectively. NM showed a relatively smaller effect size, but remained statistically significant, with an HR of 1.136.

**Table 3:** Cox regression adjusted for demographics.

Inflammation index	HR	95% CI	*p* value
SIRI	1.480	1.446–1.514	<0.001***
MLR	1.486	1.449–1.524	<0.001***
AISI	1.398	1.365–1.432	<0.001***
NM	1.136	1.101–1.172	<0.001***
MP	1.346	1.312–1.381	<0.001***
NP	1.480	1.443–1.517	<0.001***
NLR	1.489	1.456–1.524	<0.001***
SII	1.418	1.385–1.453	<0.001***
PLR	1.291	1.256–1.327	<0.001***

Model 2: adjusted for demographics, including age, gender, and race. All inflammation-derived hematological indices were winsorised at the 1st and 99th percentiles and then standardized; hazard ratios are presented per 1 SD increase. HR, hazard ratio; CI, confidence interval. *p < 0.05, **p < 0.01, ***p < 0.001.

After full adjustment for demographic characteristics, comorbidities, and laboratory variables, all nine inflammation-derived hematological indices remained independently associated with 28-day mortality, although the effect estimates were attenuated compared with those in the unadjusted and demographically adjusted models (Table 4). In the fully adjusted model, the HRs for NP, MLR, NLR, SIRI, SII, AISI, MP, PLR, and NM were 1.223, 1.232, 1.221, 1.212, 1.177, 1.167, 1.160, 1.141, and 1.068, respectively, all with *p* < 0.001.

**Table 4:** Fully adjusted cox regression.

Inflammation index	HR	95% CI	*p* value
MP	1.160	1.128–1.193	<0.001***
MLR	1.232	1.197–1.267	<0.001***
AISI	1.212	1.180–1.245	<0.001***
NLR	1.221	1.189–1.254	<0.001***
NM	1.068	1.033–1.104	<0.001***
AIRI	1.167	1.136–1.199	<0.001***
NP	1.223	1.188–1.259	<0.001***
SII	1.177	1.145–1.209	<0.001***
PLR	1.141	1.108–1.176	<0.001***

Model 3: fully adjusted for demographics and all covariates. Demographics: age, gender, race; comorbidities: AKI, CKD, COPD, hypertension, IHD, MI, heart failure, pneumonia, T2DM; laboratory tests: albumin, anion gap, creatinine, urea nitrogen, total bilirubin, glucose, sodium, potassium, chloride, total calcium, INR, AST, ALT. All inflammation-derived hematological indices were winsorised at the 1st and 99th percentiles and then standardized; hazard ratios are presented per 1 SD increase. HR, hazard ratio; CI, confidence interval. *p < 0.05, **p < 0.01, ***p < 0.001.

To further assess the potential influence of laboratory covariates with relatively high collinearity on the estimates from the fully adjusted Cox models, we constructed a reduced-collinearity model by retaining representative variables, such as retaining creatinine while removing blood urea nitrogen among renal function markers. The results (Supplementary Table S4) showed that the adjusted HRs for the nine inflammation-derived hematological indices were highly consistent with those from the complete, fully adjusted models, with changes in HRs all below 2%. All indices remained statistically significant, suggesting that the main Cox regression findings were robust to potential covariate collinearity. Overall, all nine inflammation-derived hematological indices were significantly associated with the risk of 28-day mortality in patients with sepsis across the unadjusted, demographically adjusted, and fully adjusted models.

### 3.3 ROC analysis of inflammation-derived ratios

**Figure 2:**
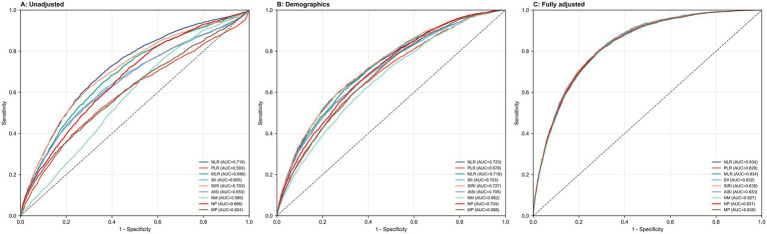
Receiver operating characteristic curves of nine inflammation-derived hematological indices for predicting 28-day mortality in patients with sepsis. The figure shows the discriminative performance of nine inflammation-derived hematological indices for 28-day mortality under different model specifications. Panel **(A)** Shows the unadjusted models, Panel **(B)** shows the models adjusted for demographic variables, and Panel **(C)** shows the fully adjusted models. In the unadjusted models, which reflect the standalone discriminative ability of each index, NLR showed the highest AUC of 0.716, followed by SIRI, MLR, and NP, with AUCs of 0.703, 0.686, and 0.666, respectively; PLR, MP, and NM showed relatively weaker discrimination. After adjustment for age, sex, and race, the AUCs increased modestly, with SIRI, NLR, and MLR showing relatively better performance. In the fully adjusted models incorporating demographic characteristics, comorbidities, and laboratory variables, the AUCs further increased to 0.827–0.835, and the differences in AUC among the nine indices became markedly smaller.

To systematically evaluate the discriminatory ability of the nine inflammation-derived hematological indices for 28-day mortality in patients with sepsis, ROC curves were constructed and compared across different adjustment models (Figure 2). In the unadjusted models, all indices showed a certain degree of discriminatory ability. NLR had the highest AUC of 0.716, followed by SIRI, MLR, and NP, with AUCs of 0.703, 0.686, and 0.666, respectively. In contrast, MP, PLR, and NM showed relatively weaker discriminatory ability, with AUCs of 0.604, 0.593, and 0.580, respectively.

After adjustment for age, sex, and race, the AUCs increased overall, with SIRI, NLR, and MLR showing relatively better performance, with AUCs of 0.727, 0.723, and 0.718, respectively. After full adjustment for demographic characteristics, comorbidities, and laboratory variables, the discriminatory ability of all models improved further, with AUCs ranging from 0.827 to 0.835. SIRI showed the highest AUC of 0.835, followed by NLR and MLR, both with an AUC of 0.834. It should be noted that the higher AUCs in the fully adjusted models reflect the overall discriminatory ability of models combining inflammation-derived hematological indices with demographic characteristics, comorbidities, and routine laboratory variables, and should not be interpreted as indicating strong predictive performance of a single inflammatory index alone. After full adjustment, the differences in AUCs among the indices became markedly smaller, suggesting that, once clinical covariates were incorporated, the incremental discriminatory differences among the nine inflammation-derived indices became more comparable.

### 3.4 RCS analysis of inflammatory indices

**Figure 3:**
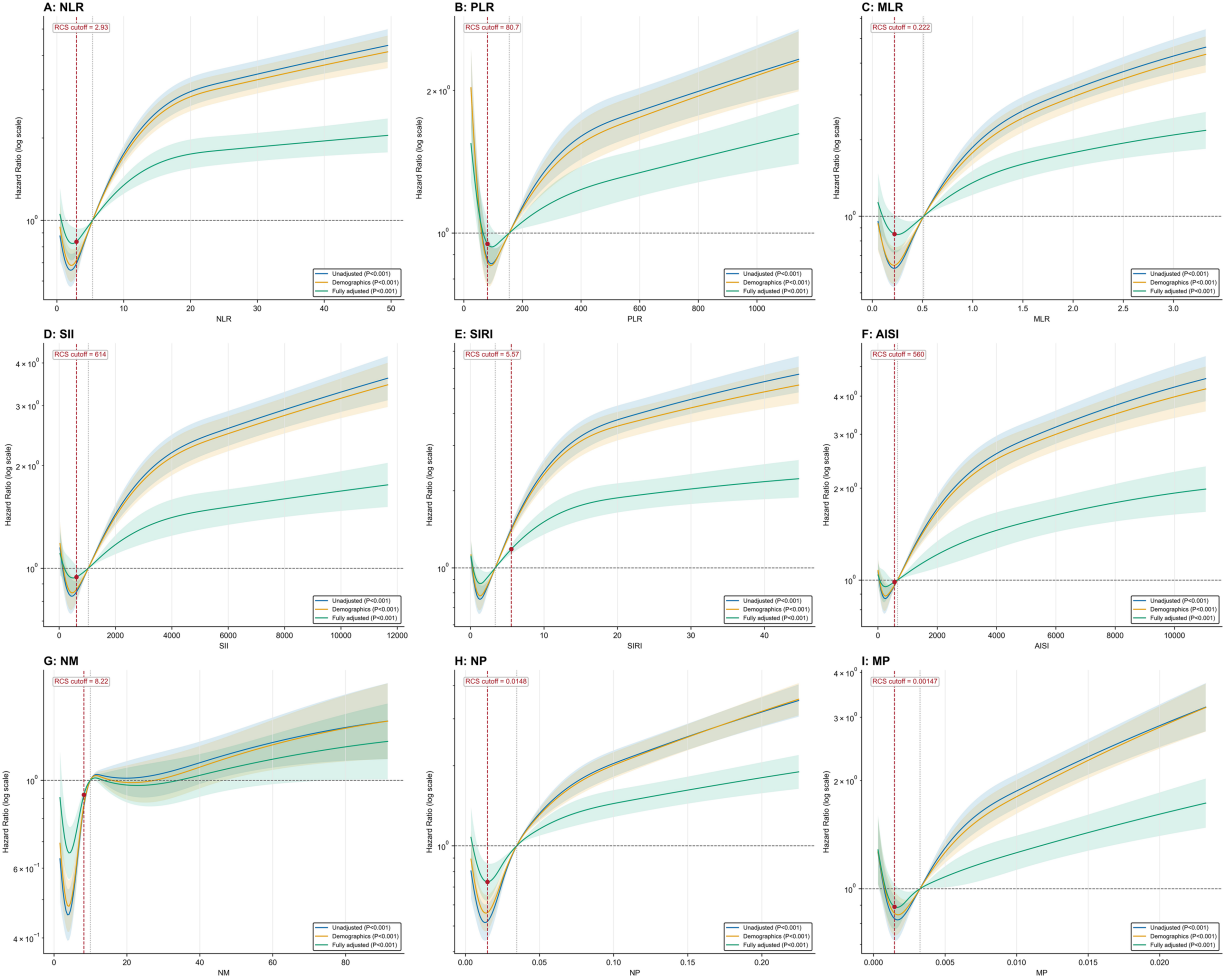
RCS analyses of the associations between nine inflammation-derived hematological indices and 28-day mortality in patients with sepsis. RCS curves were used to evaluate the potentially nonlinear associations of NLR, PLR, MLR, SII, SIRI, AISI, NM, NP, and MP with 28-day all-cause mortality after ICU admission in patients with sepsis. Panels **(A–I)** correspond to NLR, PLR, MLR, SII, SIRI, AISI, NM, NP, and MP, respectively. The *y*-axis represents the hazard ratio on a logarithmic scale, and the *x*-axis represents the level of each inflammation-derived hematological index. The blue curves represent the unadjusted models, the yellow curves represent the models adjusted for demographic variables, and the green curves represent the fully adjusted models; shaded areas indicate 95% confidence intervals. The horizontal dashed line indicates HR = 1. The red vertical dashed lines indicate the RCS-derived risk inflection reference points, which were 2.93 for NLR, 80.7 for PLR, 0.222 for MLR, 614 for SII, 5.57 for SIRI, 560 for AISI, 8.22 for NM, 0.0148 for NP, and 0.00147 for MP. All nine inflammation-derived hematological indices showed significant nonlinear associations with 28-day mortality.

To explore potential non-linear associations between inflammation-derived ratios and 28-day mortality risk in patients with sepsis, RCS analyses were performed (Figure 3). The results showed significant non-linear associations between all nine inflammation-derived indices (NLR, PLR, MLR, SII, SIRI, AISI, NP, NM, and MP) and mortality risk, with all *p* values for non-linearity <0.001.

Overall, the risk curves for most inflammation-derived indices showed a similar pattern, characterized by a decline in risk or relatively low risk at lower levels, followed by a gradual increase in risk beyond a certain inflection point. The reference inflection points identified from the RCS curves were 2.93 for NLR, 80.7 for PLR, 0.222 for MLR, 614 for SII, 5.57 for SIRI, 560 for AISI, 8.22 for NM, 0.0148 for NP, and 0.00147 for MP. Beyond these reference points, mortality risk generally increased with higher levels of most indices, particularly NLR, MLR, SII, SIRI, AISI, NP, and MP, suggesting that higher levels of these indices may be closely associated with greater mortality risk.

Across the different adjustment models, the risk curves from the unadjusted and demographically adjusted models were generally similar. After full adjustment for demographic characteristics, comorbidities, and laboratory variables, the strength of the associations was attenuated, but the overall curve patterns remained largely consistent. In particular, NLR, MLR, SIRI, SII, AISI, and NP continued to show relatively stable upward trends after full adjustment, suggesting that their non-linear associations were not fully explained by demographic factors, comorbidity burden, or routine laboratory variables. By contrast, the risk curve for NM was relatively flat, indicating that its dose–response relationship with mortality risk may be weaker.

### 3.5 Calibration curves and decision curve analysis

To further evaluate the robustness and potential clinical utility of the nine inflammation-derived hematological indices for predicting 28-day mortality in patients with sepsis, calibration curves and decision curve analyses were performed.

**Figure 4:**
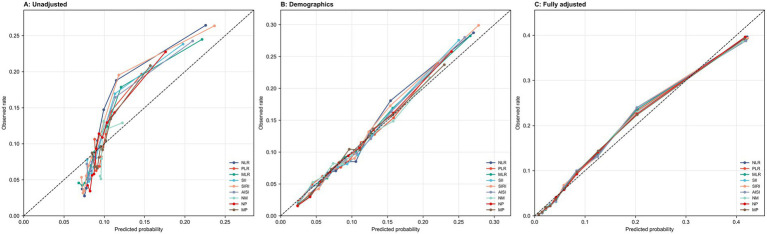
Calibration curves of nine inflammation-derived hematological indices for predicting 28-day mortality in patients with sepsis. Panels **(A–C)** represent the unadjusted, demographics-adjusted, and fully adjusted models, respectively. The *x*-axis shows the predicted probability of mortality, and the *y*-axis shows the observed mortality rate; the black dashed line indicates ideal calibration. Calibration showed some fluctuation in the unadjusted models, improved after adjustment for demographic variables, and became closer to the ideal line after further adjustment for demographic characteristics, comorbidities, and laboratory variables. The improved calibration in the fully adjusted models reflects the overall performance of models combining inflammation-derived indices with clinical covariates, rather than the independent predictive ability of the indices alone.

The calibration curves (Figure 4) showed some deviation between predicted probabilities and observed risks in the unadjusted models, particularly with noticeable fluctuations in the low predicted probability range. After adjustment for age, sex, and race, model calibration improved. After full adjustment for demographic characteristics, comorbidities, and laboratory variables, the calibration curves of the index-based models were generally closer to the ideal reference line, and the curves for different indices were highly similar, indicating good calibration consistency after integration of clinical covariates.

**Figure 5:**
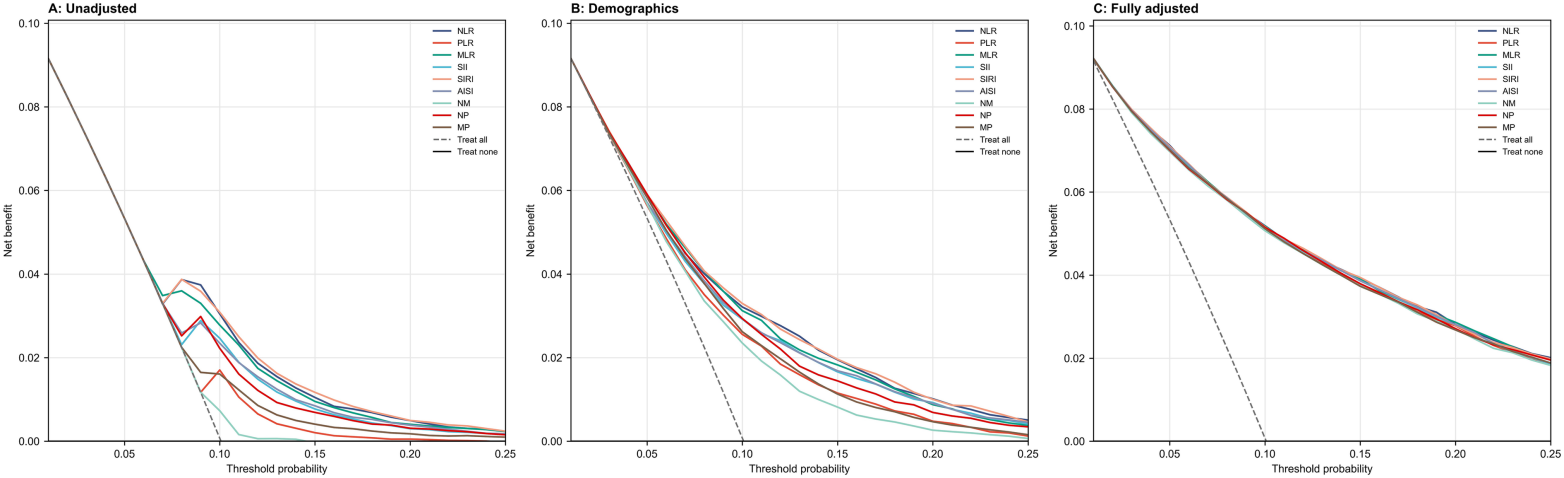
Decision curve analysis of nine inflammation-derived hematological indices for predicting 28-day mortality in patients with sepsis. Panels **(A–C)** represent the unadjusted, demographics-adjusted, and fully adjusted models, respectively. The *x*-axis shows the threshold probability, and the *y*-axis shows the net benefit; the grey dashed line represents the treat-all strategy, and the black solid line represents the treat-none strategy. In the unadjusted and demographics-adjusted models, the inflammation-derived hematological indices provided net benefit only across part of the threshold-probability range. After full adjustment for demographic characteristics, comorbidities, and laboratory variables, all models showed relatively stable net benefit across the main threshold-probability range, and the decision curves largely overlapped, suggesting limited differences in incremental clinical utility among the indices after incorporation of clinical covariates.

Decision curve analysis (Figure 5) showed that, in the unadjusted and demographically adjusted models, the inflammation-derived index models provided net benefit over the treat-all and treat-none strategies only within certain threshold probability ranges. After full adjustment, the net benefit range expanded, and all nine indices outperformed both the treat-all and treat-none strategies across threshold probabilities of 1 to 25% (Supplementary Table S5). At clinically relevant thresholds of 5, 10, 15, 20, and 25%, the net benefit values were similar across models, consistent with the substantial overlap of the decision curves in the figure. Overall, inflammation-derived hematological indices may provide some clinical net benefit when combined with clinical covariates, but the differences in incremental clinical utility among the indices were limited.

### 3.6 Incremental predictive value beyond the SOFA score

**Figure 6:**
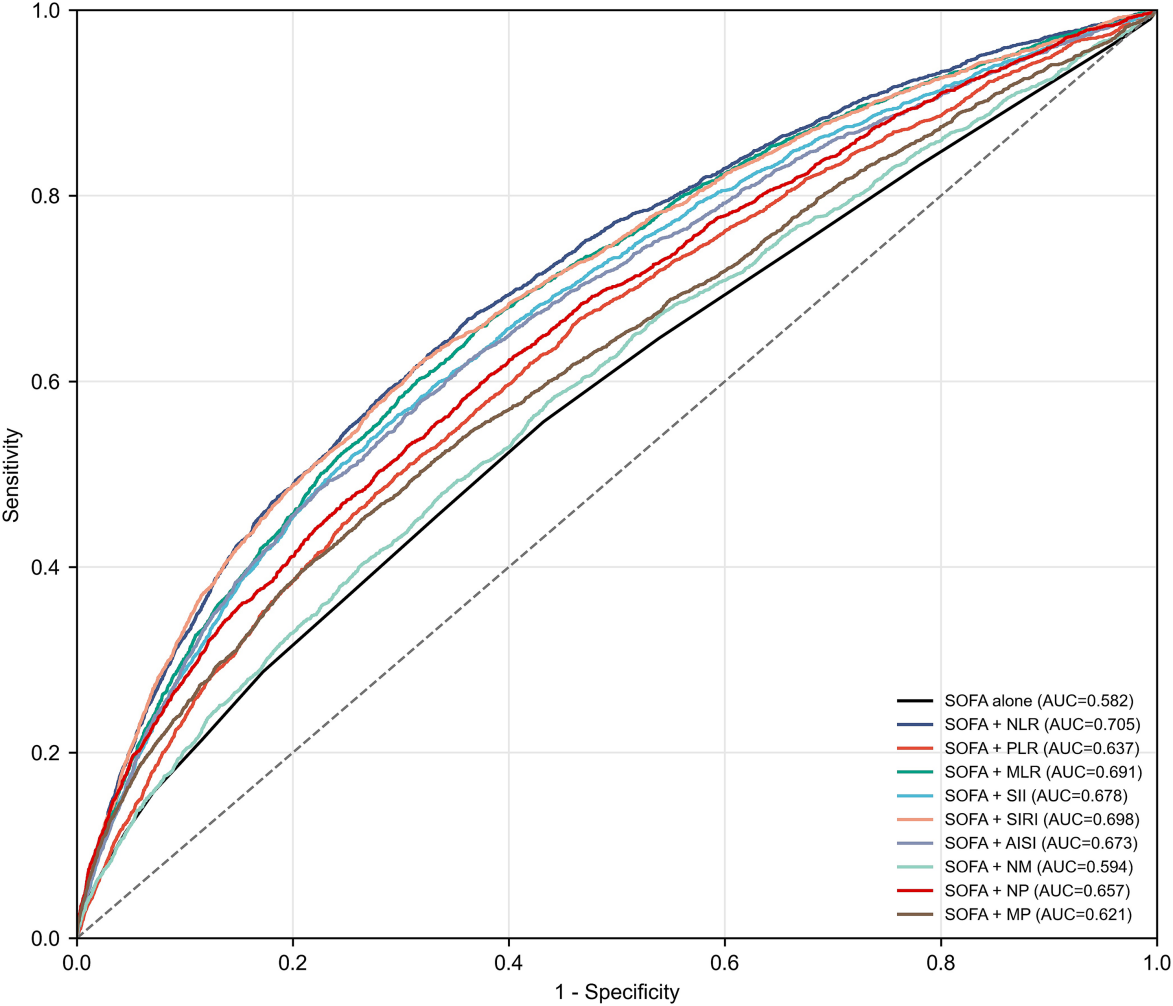
ROC curves of SOFA combined with nine inflammation-derived hematological indices for predicting 28-day mortality in patients with sepsis. This figure compares the discriminative performance of the SOFA-alone model and SOFA-based models incorporating each inflammation-derived hematological index. The AUC of the SOFA-alone model was 0.582. After adding the indices, all combined models showed varying degrees of improvement, with SOFA + NLR showing the highest AUC of 0.705, followed by SOFA + SIRI and SOFA + MLR, with AUCs of 0.698 and 0.691, respectively. These findings suggest that NLR, SIRI, and MLR provide relatively greater complementary discriminative information beyond SOFA, but should not be interpreted as evidence that any single index can replace the SOFA score.

To evaluate the incremental predictive value of inflammation-derived hematological indices beyond the SOFA score, we compared the discriminatory ability of the SOFA-only model with that of models combining SOFA with each inflammatory index for predicting 28-day mortality after the landmark time. The AUC of the SOFA-only model was 0.582. After adding inflammation-derived hematological indices, the AUCs of all combined models increased. The SOFA plus NLR model had the highest AUC of 0.705, followed by SOFA plus SIRI and SOFA plus MLR, with AUCs of 0.698 and 0.691, respectively. The AUCs for SOFA plus SII, SOFA plus AISI, and SOFA plus NP were 0.678, 0.673, and 0.657, respectively, whereas the improvements for SOFA plus PLR, SOFA plus MP, and SOFA plus NM were relatively limited, with AUCs of 0.637, 0.621, and 0.594, respectively. Overall, NLR, SIRI, and MLR showed relatively greater incremental discriminatory value beyond SOFA, suggesting that they may serve as supplementary risk markers to conventional severity scores (Figure 6).

Further incremental predictive analyses (Supplementary Table S9) showed that, compared with the SOFA-only model, all models combining SOFA with one of the nine inflammation-derived hematological indices achieved significantly higher AUCs. The largest ΔAUC was observed for SOFA plus NLR, at 0.123, followed by SOFA plus SIRI and SOFA plus MLR, with ΔAUCs of 0.116 and 0.109, respectively. Likelihood ratio tests for nested Cox models showed that all combined models significantly improved model fit compared with the SOFA-only model. The integrated discrimination improvement and continuous net reclassification improvement analyses further supported these findings, with relatively greater reclassification improvement observed for SOFA plus SIRI, SOFA plus NLR, and SOFA plus MLR. These results suggest that SIRI, NLR, and MLR may provide relatively stronger supplementary predictive information beyond the SOFA score.

### 3.7 External validation

The external validation cohort included 850 patients, of whom 182 died within 28 days after ICU admission (Supplementary Table S2). Non-survivors were substantially older, but most comorbidities, disease severity scores, and inflammation-derived hematological indices did not differ significantly between survivors and non-survivors. Compared with the development cohort, the between-group differences in the nine inflammation-derived hematological indices were generally weaker in the external validation cohort, with only SII and PLR showing borderline differences. These findings suggest potential heterogeneity across cohorts in the baseline distributions of inflammation-derived indices and their crude associations with mortality risk.

Further Cox regression analysis showed that the prognostic associations of the nine inflammation-derived hematological indices were substantially attenuated in the external validation cohort compared with the development cohort (Table 5). In the unadjusted models, only SII and PLR were significantly associated with the risk of 28-day mortality after the landmark time, with HRs of 1.175 and 1.165, respectively. After adjustment for age, sex, and race, SII and PLR retained weak statistical associations, with HRs of 1.133 and 1.129, respectively. However, after full adjustment for demographic characteristics, comorbidities, and laboratory variables, none of the nine inflammation-derived hematological indices remained significantly associated with mortality risk.

**Table 5:** Cox regression analysis of nine inflammation-derived hematological indices for landmark-based 28-day mortality in the external validation cohort.

Inflammation index	Unadjusted HR (95% CI)	Unadjusted *p* value	Adjusted for demographics HR (95% CI)	Adjusted for demographics *p* value	Fully adjusted HR (95% CI)	Fully adjusted *p* value
MP	0.981 (0.839–1.147)	0.814	0.986 (0.845–1.151)	0.860	1.014 (0.874–1.177)	0.850
MLR	1.020 (0.885–1.176)	0.783	1.049 (0.908–1.212)	0.516	1.064 (0.918–1.233)	0.410
SIRI	1.013 (0.880–1.165)	0.861	1.025 (0.889–1.182)	0.736	1.028 (0.886–1.192)	0.717
NLR	1.051 (0.914–1.208)	0.484	1.045 (0.909–1.202)	0.537	1.015 (0.880–1.172)	0.833
NM	0.964 (0.827–1.123)	0.639	0.965 (0.827–1.125)	0.649	0.955 (0.812–1.123)	0.574
AISI	1.092 (0.965–1.236)	0.162	1.076 (0.949–1.219)	0.252	1.055 (0.923–1.205)	0.434
NP	1.019 (0.883–1.176)	0.798	1.015 (0.881–1.168)	0.838	1.032 (0.899–1.184)	0.654
SII	1.175 (1.042–1.326)	0.009**	1.133 (1.005–1.278)	0.042*	1.073 (0.944–1.220)	0.280
PLR	1.165 (1.034–1.312)	0.012*	1.129 (1.001–1.273)	0.049*	1.066 (0.936–1.214)	0.334

All inflammation-derived hematological indices are winsorised at the 1st and 99th percentiles and then standardized; hazard ratios are presented per 1 SD increase. The unadjusted model included each index alone. The demographic model was adjusted for age, gender, and race. The fully adjusted model was further adjusted for AKI, CKD, COPD, hypertension, IHD, MI, heart failure, pneumonia, T2DM, albumin, anion gap, creatinine, urea nitrogen, total bilirubin, glucose, sodium, potassium, chloride, total calcium, INR, AST, and ALT. HR, hazard ratio; CI, confidence interval. *p < 0.05 and **p < 0.01.

### 3.8 Sensitivity analyses

To assess the robustness of the main Cox regression findings, two sensitivity analyses were further performed. First, the fully adjusted Cox regression results from the multiply imputed dataset were compared with those from the complete-case dataset. The results showed that all nine inflammation-derived hematological indices were significantly associated with the risk of 28-day mortality after the landmark time in both datasets, with only small changes in HRs. The largest changes were observed for AISI and SIRI, at 2.5 and 2.4%, respectively, suggesting that the main findings were not substantially affected by the missing data imputation strategy (Supplementary Table S6).

Second, sensitivity analysis of outlier handling showed that, both before and after winsorization, all nine inflammation-derived hematological indices retained the same direction of association and remained statistically significant. Notably, the HRs for NLR, PLR, SII, and NP changed by more than 10%, indicating that outlier handling affected the effect size estimates for some indices. However, this did not alter the main conclusion that these indices were significantly associated with mortality risk overall (Supplementary Table S8).

## 4 Discussion

Using the large MIMIC-IV database, this study systematically compared the associations between nine inflammation-derived hematological indices and the risk of 28-day mortality in 26,512 patients with sepsis from the MIMIC-IV database, and further evaluated their generalizability in an independent external validation cohort. In the development cohort, all nine inflammation-derived hematological indices were statistically associated with 28-day mortality and remained significant across different levels of adjustment. Among them, MLR, NP, NLR, and SIRI showed relatively larger effect estimates in the fully adjusted models. However, the overall effect sizes of these associations were limited, and the discriminatory ability of the inflammation-derived indices alone was only moderate. In the unadjusted ROC analysis, the best-performing index was NLR, with an AUC of 0.716, followed by SIRI and MLR, with AUCs of 0.703 and 0.686, respectively. These findings suggest that a single inflammatory index is unlikely to serve as a high-precision standalone predictive tool. Although the AUCs increased substantially in the fully adjusted models, this improvement mainly reflected the contribution of clinical covariates, such as age, comorbidities, and laboratory variables, rather than the predictive capacity of the inflammatory indices themselves. Overall, our findings support the interpretation of inflammation-derived hematological indices as candidate adjunctive biomarkers for early risk stratification in sepsis, rather than as strong independent predictive tools.

The external validation results provide an important qualification to the conclusions of this study. In contrast to the development cohort, in which all nine inflammation-derived hematological indices were significantly associated with 28-day mortality, these associations were substantially attenuated in the external validation cohort and were no longer statistically significant after full adjustment for demographic characteristics, comorbidities, and laboratory variables. This finding suggests that the risk associations observed in the development cohort may not be consistently transferable to independent clinical populations, and that the cross-cohort generalizability of these nine indices remains limited. Potential explanations for this discrepancy include heterogeneity across cohorts in population characteristics, comorbidity profiles, sources of infection, disease severity, ICU admission criteria, treatment strategies, laboratory testing procedures, and sample size. In particular, the relatively limited sample size and number of death events in the external validation cohort may have further reduced statistical power in the fully adjusted models. Therefore, the external validation results require cautious interpretation of the predictive value of these indices. They may be more appropriately regarded as candidate adjunctive risk markers, rather than independent predictive tools with stable external reproducibility.

From a pathophysiological perspective, inflammation-derived hematological indices may reflect the integrated state of inflammatory activation, immunosuppression, and platelet-related immunothrombotic responses during sepsis. Sepsis is not merely a process of excessive inflammation, but a complex syndrome characterized by interactions among innate immune activation, adaptive immune suppression, endothelial injury, and coagulation abnormalities (12, 15). Previous studies have shown that neutrophilia and lymphocyte apoptosis are important manifestations of early immune dysregulation in sepsis (16, 17), whereas monocyte dysfunction and decreased human leukocyte antigen-DR (HLA-DR) expression are closely associated with immunosuppression, secondary infection, and poor outcomes (18). For example, a prospective cohort study found that patients with sepsis often presented with markedly increased neutrophil counts and apoptosis of lymphocyte subsets within 48 h of onset, which were independently associated with short-term mortality (16). In addition, several studies have shown that persistently low monocyte HLA-DR expression is an important marker of immunosuppression in sepsis and is significantly associated with an increased risk of secondary infection and higher 28-day and 90-day mortality (19, 20).

Furthermore, our findings are consistent with the recognized immune heterogeneity of sepsis. Previous transcriptomic and immunophenotyping studies have shown that patients with sepsis may simultaneously or sequentially exhibit neutrophil-mediated hyperinflammation, lymphocyte functional exhaustion, and impaired monocyte antigen presentation (12, 21). Therefore, composite inflammation-derived hematological indices, such as NLR, SIRI, MLR, and the systemic immune-inflammation index (SII), may better capture the imbalance between inflammatory activation and immunosuppression than individual blood cell counts by integrating relative changes across different immune cell populations (22). In the present study, RCS analysis showed significant non-linear associations between all nine inflammation-derived hematological indices and the risk of 28-day mortality after the landmark time, indicating that their prognostic implications may not follow a simple linear pattern and may involve certain risk transition ranges. This finding suggests that clinical interpretation of these indices should consider not only their average effects as continuous variables, but also their potential dose–response patterns. However, the cut-off values identified in this study were primarily used to describe risk transition patterns in the RCS curves and should not be directly interpreted as thresholds for clinical diagnosis, intervention, or treatment decisions. Further validation in independent prospective cohorts is needed to determine the stability and clinical applicability of these risk transition points.

From a clinical perspective, inflammation-derived hematological indices are derived from routine complete blood count tests and have the advantages of rapid availability, low cost, reproducibility, and suitability for dynamic monitoring. They may therefore have practical potential in time-sensitive settings such as emergency departments and ICUs (22). Nevertheless, our findings indicate that their predictive utility should be interpreted cautiously. When used alone, these indices showed only moderate discriminatory ability overall. The higher AUCs observed in the fully adjusted models largely reflected the contribution of clinical covariates, including age, comorbidities, and routine laboratory variables, rather than the independent predictive performance of the inflammatory indices themselves. The SOFA combined analysis and decision curve analysis suggested that inflammation-derived hematological indices may provide some supplementary information when combined with conventional severity scores or clinical covariates, with NLR, SIRI, and MLR showing relatively stronger performance. However, this improvement mainly represents statistical enhancement at the model level and does not imply that any single inflammatory index can replace comprehensive scoring systems such as SOFA or APACHE II, or directly guide antimicrobial therapy and organ support strategies. Therefore, these indices are more appropriately considered as adjunctive variables in clinical risk assessment, and their practical clinical value requires further validation in multicenter prospective studies.

The selection of the study population has important implications for the applicability of our findings. We excluded patients with severe liver failure, end-stage renal disease, malignancy, or immunodeficiency-related diagnoses, mainly to reduce the influence of highly heterogeneous underlying diseases on the interpretation of inflammation-derived hematological indices. These conditions can substantially alter peripheral blood cell composition, bone marrow hematopoiesis, immune-inflammatory status, coagulation function, chronic organ reserve, and short-term mortality risk. For example, malignancy and its related treatments may lead to bone marrow suppression and abnormal leukocyte profiles (23); immunodeficiency may weaken or alter the host response to infection (24); and end-stage renal disease and severe liver failure are often accompanied by chronic inflammation, coagulation abnormalities, metabolic disturbances, and reduced organ reserve (25, 26). Therefore, this exclusion strategy helped improve the homogeneity of the study population and the internal interpretability of comparisons among different indices. However, it also limits the generalizability of the findings to complex real-world ICU patients with sepsis. Given that these high-risk conditions are not uncommon in clinical sepsis populations and are often accompanied by complex immune-inflammatory phenotypes, multiple comorbidities, and higher mortality risk, our findings should primarily be applied to a relatively selected ICU sepsis population. They should not be directly generalized to patients with complex multimorbidity, markedly abnormal immune function, or end-stage chronic organ failure. In addition, these exclusion criteria also limit the interpretation of the CCI and APACHE II scores in the baseline characteristics. Because malignancy, end-stage renal disease, severe liver failure, and immune dysfunction-related diseases carry substantial weight or have important effects in scoring systems such as the CCI and APACHE II, the CCI and APACHE II scores reported in this study were calculated within a relatively restricted range of comorbidity burden and disease severity. They are therefore more appropriate for describing baseline differences within the selected study cohort, rather than as a complete reflection of the overall disease burden among complex real-world patients with sepsis and multimorbidity.

In addition to these applicability issues, several limitations should be acknowledged. First, this was a retrospective observational study. Although it was based on a large development cohort and included external validation, multivariable adjustment, and sensitivity analyses, residual confounding, information bias, and selection bias could not be fully eliminated. Second, the development cohort was derived from MIMIC-IV, whereas the external validation cohort was obtained from a single-center Chinese hospital. Differences in population characteristics, comorbidity profiles, laboratory testing procedures, and clinical management strategies between cohorts may partly explain the attenuation of the effects of some indices in external validation. Third, this study used only early hematological variables obtained within the first 24 h after ICU admission to calculate the nine inflammation-derived indices, and did not include subsequent serial monitoring data or dynamic trajectory information. Sepsis is a highly dynamic syndrome, and host inflammatory responses, immunosuppressive states, and blood cell composition may change substantially with infection control, organ support therapy, disease progression, and the development of complications. Therefore, indices measured within a single early time window can only reflect the inflammatory and immune status at the early stage of ICU admission, and cannot assess the temporal characteristics, trajectory subtypes, or dynamic prognostic relationships of inflammation-derived hematological indices over the disease course. Fourth, although we adjusted for multiple clinical confounders, the study lacked deeper information on cytokines, immunophenotypes, pathogen characteristics, infection sites, and treatment responses. Therefore, the mechanisms underlying these inflammation-derived indices could not be further elucidated. Future studies should incorporate complete blood count data from multiple time points to further explore whether trends, persistent abnormalities, and recovery patterns in these indices can provide additional prognostic information.

Future research should further validate the prognostic performance and cross-cohort stability of inflammation-derived hematological indices in large, multicenter, prospective cohorts, particularly those including patients with complex multimorbidity and immune dysfunction. In addition, future studies may integrate longitudinal complete blood count data, conventional scoring systems, pathogen information, cytokines, transcriptomic data, and immunophenotyping data to develop more refined multimodal risk assessment models and to further evaluate their practical value in early risk stratification, resource allocation, and clinical decision-making in emergency and ICU settings.

## 5 Conclusion

In this study, all nine inflammation-derived hematological indices were statistically associated with the risk of 28-day mortality in patients with sepsis in the development cohort and showed certain non-linear risk patterns. However, when used alone, these indices had limited discriminatory ability overall and demonstrated only moderate predictive performance. NLR, SIRI, MLR, and NP showed relatively stronger risk associations or supplementary predictive information in some analyses, but these associations were not consistently maintained after full adjustment in the external validation cohort, indicating limited cross-cohort generalizability. Therefore, inflammation-derived hematological indices may be more appropriately used as supplementary information alongside conventional severity scores and clinical risk assessment, rather than as independent tools for mortality risk prediction. Their practical clinical utility requires further validation in large, multicenter, prospective studies.

## Data Availability

The MIMIC-IV data used in this study are publicly available from PhysioNet to credentialed users. The institutional cohort data are not publicly available due to privacy and ethical restrictions. De-identified data may be obtained from the corresponding author upon reasonable request and subject to approval by the relevant ethics committee.
