# Supplementary material for: Comparative predictive value of nine inflammation-derived haematological indices for 28-day mortality in patients with sepsis: a multicentre retrospective cohort study
Source: Front Med (Lausanne). 2026 Jun 19;13:1857973. doi: 10.3389/fmed.2026.1857973 (PMC13328474; doi:10.3389/fmed.2026.1857973)
Supplement: Supplementary file 1 [file Data_Sheet_1.ZIP › Supplementary Files/Supplementary Table S1.docx]

**Supplementary Table S1. Missingness of original variables across sample-selection stages**

| **Variable** | **Minimum Missing (%)** |
| --- | --- |
| Age | 0 |
| Sex | 0 |
| Race | 0 |
| Hypertension | 0 |
| Acute kidney injury | 0 |
| Pneumonia | 0 |
| Cerebrovascular disease | 0 |
| Chronic kidney disease | 0 |
| Type 2 diabetes mellitus | 0 |
| Type 1 diabetes mellitus | 0 |
| Hyperlipidemia | 0 |
| Heart failure | 0 |
| Myocardial infarction | 0 |
| Ischemic heart disease | 0 |
| Chronic obstructive pulmonary disease | 0 |
| Admission time | 0 |
| Discharge time | 0 |
| Hospital length of stay, days | 0 |
| All-cause death indicator | 0 |
| In-hospital death indicator | 0 |
| First absolute lymphocyte count | 0 |
| First platelet count | 0 |
| First absolute neutrophil count | 0 |
| First absolute monocyte count | 0 |
| First red blood cell count | 0 |
| First white blood cell count | 0 |
| first_nbps | 0 |
| first_nbpd | 0 |
| first_nbpm | 0 |
| first_hr | 0 |
| first_rr | 0 |
| First hemoglobin | 0.1 |
| First hematocrit | 0.1 |
| First red cell distribution width | 0.1 |
| First creatinine | 0.1 |
| First anion gap | 0.2 |
| First chloride | 0.2 |
| First sodium | 0.2 |
| First blood urea nitrogen | 0.2 |
| First glucose | 0.3 |
| First potassium | 0.3 |
| sofa | 1.2 |
| first_temperaturef | 1.3 |
| apacheii | 1.8 |
| apsiii | 2.5 |
| First total calcium | 2.8 |
| sirs | 3.4 |
| sapsii | 3.4 |
| oasis | 8.7 |
| gcs | 9.7 |
| first_lactate | 11.2 |
| first_ph | 11.2 |
| first_po2 | 11.2 |
| first_calculated_total_co2 | 11.2 |
| first_pco2 | 11.2 |
| first_spo2 | 12.3 |
| charlson | 13.5 |
| first_lactate_dehydrogenase_ld | 13.6 |
| First albumin | 14.7 |
| First international normalized ratio | 14.9 |
| First activated partial thromboplastin time | 16.9 |
| First aspartate aminotransferase | 21.5 |
| First alanine aminotransferase | 22.8 |
| First total bilirubin | 24.3 |
| first_free_calcium | 63.3 |
| first_fibrinogen_functional | 66.6 |
| first_creatine_kinase_ck | 74.4 |
| first_triglycerides | 79.9 |
| first_creactive_protein | 82.6 |
| first_troponin_t | 82.8 |
| first_cholesterol_total | 87.3 |
| first_highsensitivity_crp | 99.9 |
