# Supplementary material for: Comparative predictive value of nine inflammation-derived haematological indices for 28-day mortality in patients with sepsis: a multicentre retrospective cohort study
Source: Front Med (Lausanne). 2026 Jun 19;13:1857973. doi: 10.3389/fmed.2026.1857973 (PMC13328474; doi:10.3389/fmed.2026.1857973)
Supplement: Supplementary file 1 [file Data_Sheet_1.ZIP › Supplementary Files/Supplementary Table S2.docx]

**Supplementary Table S2. Baseline Characteristics of the External Validation Cohort Stratified by Landmark-Based 28-Day Mortality**

| **Variable** | **Overall (N = 850)** | **Survivor (N = 668)** | **No-survivor (N = 182)** | **P-value** | **SMD** |
| --- | --- | --- | --- | --- | --- |
| **characteristics** |  |  |  |  |  |
| Age | 64.52 ± 17.17 | 62.69 ± 17.31 | 71.25 ± 14.82 | <0.001 | 0.531 |
| MAP | 81.14 ± 15.63 | 81.14 ± 15.67 | 81.13 ± 15.53 | 0.992 | 0.001 |
| RR | 19.84 ± 5.71 | 19.76 ± 5.61 | 20.12 ± 6.09 | 0.485 | 0.060 |
| Gender |  |  |  | 0.625 | 0.048 |
| F | 617 (72.59%) | 488 (73.05%) | 129 (70.88%) |  |  |
| M | 233 (27.41%) | 180 (26.95%) | 53 (29.12%) |  |  |
| Race |  |  |  |  |  |
| other | 850 (100.00%) | 668 (100.00%) | 182 (100.00%) |  |  |
| **Comorbidities** |  |  |  |  |  |
| HTN, n (%) |  |  |  | 0.417 | 0.075 |
| No | 501 (58.94%) | 399 (59.73%) | 102 (56.04%) |  |  |
| Yes | 349 (41.06%) | 269 (40.27%) | 80 (43.96%) |  |  |
| AKI, n (%) |  |  |  | 0.149 | 0.127 |
| No | 604 (71.06%) | 483 (72.31%) | 121 (66.48%) |  |  |
| Yes | 246 (28.94%) | 185 (27.69%) | 61 (33.52%) |  |  |
| PNA, n (%) |  |  |  | 0.925 | 0.025 |
| No | 34 (4.00%) | 26 (3.89%) | 8 (4.40%) |  |  |
| Yes | 816 (96.00%) | 642 (96.11%) | 174 (95.60%) |  |  |
| CVA, n (%) |  |  |  | 0.546 | 0.058 |
| No | 523 (61.53%) | 407 (60.93%) | 116 (63.74%) |  |  |
| Yes | 327 (38.47%) | 261 (39.07%) | 66 (36.26%) |  |  |
| CKD, n (%) |  |  |  | 0.845 | 0.028 |
| No | 765 (90.00%) | 600 (89.82%) | 165 (90.66%) |  |  |
| Yes | 85 (10.00%) | 68 (10.18%) | 17 (9.34%) |  |  |
| T2DM, n (%) |  |  |  | 0.079 | 0.159 |
| No | 622 (73.18%) | 479 (71.71%) | 143 (78.57%) |  |  |
| Yes | 228 (26.82%) | 189 (28.29%) | 39 (21.43%) |  |  |
| T1DM, n (%) |  |  |  | 1.000 | 0.055 |
| No | 849 (99.88%) | 667 (99.85%) | 182 (100.00%) |  |  |
| Yes | 1 (0.12%) | 1 (0.15%) | 0 (0.00%) |  |  |
| HLD, n (%) |  |  |  | 0.006 | 0.294 |
| No | 803 (94.47%) | 623 (93.26%) | 180 (98.90%) |  |  |
| Yes | 47 (5.53%) | 45 (6.74%) | 2 (1.10%) |  |  |
| HF, n (%) |  |  |  | 0.713 | 0.038 |
| No | 614 (72.24%) | 485 (72.60%) | 129 (70.88%) |  |  |
| Yes | 236 (27.76%) | 183 (27.40%) | 53 (29.12%) |  |  |
| MI, n (%) |  |  |  | 0.174 | 0.121 |
| No | 812 (95.53%) | 642 (96.11%) | 170 (93.41%) |  |  |
| Yes | 38 (4.47%) | 26 (3.89%) | 12 (6.59%) |  |  |
| IHD, n (%) |  |  |  | 0.264 | 0.101 |
| No | 725 (85.29%) | 575 (86.08%) | 150 (82.42%) |  |  |
| Yes | 125 (14.71%) | 93 (13.92%) | 32 (17.58%) |  |  |
| COPD, n (%) |  |  |  | 0.693 | 0.070 |
| No | 841 (98.94%) | 660 (98.80%) | 181 (99.45%) |  |  |
| Yes | 9 (1.06%) | 8 (1.20%) | 1 (0.55%) |  |  |
| **Laboratory tests** |  |  |  |  |  |
| Lymphocyte count | 1.71 ± 14.04 | 1.82 ± 15.58 | 1.33 ± 5.53 | 0.503 | 0.042 |
| Neutrophil count | 11.94 ± 15.51 | 11.93 ± 16.12 | 11.95 ± 13.09 | 0.990 | 0.001 |
| Monocyte count | 0.89 ± 2.65 | 0.91 ± 2.78 | 0.83 ± 2.07 | 0.692 | 0.030 |
| PLT | 164.74 ± 124.26 | 158.23 ± 117.55 | 188.60 ± 144.13 | 0.009 | 0.231 |
| Hematocrit | 28.09 ± 12.74 | 28.03 ± 12.38 | 28.29 ± 14.03 | 0.823 | 0.019 |
| Hemoglobin | 88.33 ± 26.68 | 87.98 ± 26.20 | 89.63 ± 28.40 | 0.483 | 0.060 |
| RDW | 0.17 ± 0.04 | 0.17 ± 0.04 | 0.17 ± 0.04 | 0.778 | 0.023 |
| RBC | 3.13 ± 0.99 | 3.11 ± 0.97 | 3.20 ± 1.04 | 0.309 | 0.087 |
| WBC | 14.66 ± 22.41 | 14.77 ± 24.14 | 14.25 ± 14.36 | 0.711 | 0.026 |
| Albumin | 29.59 ± 6.07 | 29.62 ± 6.30 | 29.48 ± 5.16 | 0.756 | 0.025 |
| Anion gap | 13.02 ± 6.27 | 13.29 ± 6.12 | 12.02 ± 6.73 | 0.022 | 0.198 |
| PTT | 37.58 ± 14.86 | 37.73 ± 14.74 | 37.06 ± 15.33 | 0.599 | 0.045 |
| Creatinine | 196.61 ± 184.00 | 202.94 ± 188.39 | 173.38 ± 165.30 | 0.039 | 0.167 |
| pH | 7.14 ± 1.30 | 7.16 ± 1.26 | 7.09 ± 1.43 | 0.587 | 0.047 |
| Lac | 2.64 ± 3.10 | 2.73 ± 3.21 | 2.31 ± 2.62 | 0.072 | 0.142 |
| **Inflammation indices** |  |  |  |  |  |
| SIRI | 14.31 ± 30.51 | 14.56 ± 33.09 | 13.41 ± 18.19 | 0.535 | 0.043 |
| MP | 0.02 ± 0.09 | 0.01 ± 0.08 | 0.02 ± 0.13 | 0.785 | 0.025 |
| MLR | 1.11 ± 2.04 | 1.11 ± 2.08 | 1.11 ± 1.89 | 0.958 | 0.004 |
| NP | 0.17 ± 0.50 | 0.17 ± 0.52 | 0.17 ± 0.43 | 0.961 | 0.004 |
| NM | 34.26 ± 62.59 | 34.93 ± 66.55 | 31.83 ± 45.26 | 0.464 | 0.054 |
| NLR | 20.24 ± 22.59 | 20.11 ± 23.17 | 20.72 ± 20.38 | 0.729 | 0.028 |
| AISI | 2232.16 ± 4398.79 | 2108.89 ± 4118.74 | 2684.60 ± 5290.05 | 0.175 | 0.121 |
| SII | 3206.58 ± 4970.42 | 3031.89 ± 4843.10 | 3847.73 ± 5377.71 | 0.065 | 0.159 |
| PLR | 296.24 ± 404.28 | 283.29 ± 401.99 | 343.78 ± 410.22 | 0.078 | 0.149 |
| **Scores** |  |  |  |  |  |
| SOFA | 6.56 ± 3.54 | 6.65 ± 3.55 | 6.26 ± 3.47 | 0.192 | 0.109 |
| APSIII | 51.97 ± 21.81 | 52.17 ± 22.08 | 51.24 ± 20.83 | 0.597 | 0.044 |
| SAPSII | 41.04 ± 14.09 | 41.22 ± 13.99 | 40.39 ± 14.48 | 0.490 | 0.058 |
| Charlson | 5.18 ± 2.92 | 5.17 ± 2.93 | 5.21 ± 2.90 | 0.848 | 0.016 |
| APACHEII | 20.97 ± 7.30 | 20.93 ± 7.19 | 21.11 ± 7.71 | 0.775 | 0.024 |

*HTN, hypertension; AKI, acute kidney injury; PNA, pneumonia; CVA, cerebrovascular accident; CKD, chronic kidney disease; T2DM, type 2 diabetes mellitus; T1DM, type 1 diabetes mellitus; HLD, hyperlipidemia; HF, heart failure; MI, myocardial infarction; IHD, ischemic heart disease; COPD, chronic obstructive pulmonary disease; RBC, red blood cell; WBC, white blood cell; RDW, red cell distribution width; PLT, platelet count; NLR, neutrophil-to-lymphocyte ratio; PLR, platelet-to-lymphocyte ratio; SII, systemic immune-inflammation index; SIRI, systemic inflammation response index; MLR, monocyte-to-lymphocyte ratio; NP, neutrophil-to-platelet ratio; NM, neutrophil-to-monocyte ratio; MP, monocyte-to-platelet ratio; APSIII, acute physiology score III; SAPSII, simplified acute physiology score II; SOFA, sequential organ failure assessment; APACHEII, acute physiology and chronic health evaluation II; MAP, mean arterial pressure; RR, respiratory rate; Lac, lactate; PTT, partial thromboplastin time; SMD, standardized mean difference.
