# Supplementary material for: Comparative predictive value of nine inflammation-derived haematological indices for 28-day mortality in patients with sepsis: a multicentre retrospective cohort study
Source: Front Med (Lausanne). 2026 Jun 19;13:1857973. doi: 10.3389/fmed.2026.1857973 (PMC13328474; doi:10.3389/fmed.2026.1857973)
Supplement: Supplementary file 1 [file Data_Sheet_1.ZIP › Supplementary Files/Supplementary Table S3-1.docx]

**Supplementary Table S3-1. Variance Inflation Factor Analysis for Covariates in the Fully Adjusted Cox Model**

| **Cohort** | **Model** | **Variable** | **VIF** | **Interpretation** |
| --- | --- | --- | --- | --- |
| Derivation cohort | Fully adjusted covariates | race_WHITE | 2.776 | No harmful collinearity |
| Derivation cohort | Fully adjusted covariates | race_OTHER | 2.736 | No harmful collinearity |
| Derivation cohort | Fully adjusted covariates | creatinine | 2.689 | No harmful collinearity |
| Derivation cohort | Fully adjusted covariates | urea_nitrogen | 2.310 | No harmful collinearity |
| Derivation cohort | Fully adjusted covariates | chloride | 2.105 | No harmful collinearity |
| Derivation cohort | Fully adjusted covariates | sodium | 1.935 | No harmful collinearity |
| Derivation cohort | Fully adjusted covariates | AST | 1.887 | No harmful collinearity |
| Derivation cohort | Fully adjusted covariates | ALT | 1.775 | No harmful collinearity |
| Derivation cohort | Fully adjusted covariates | CKD | 1.704 | No harmful collinearity |
| Derivation cohort | Fully adjusted covariates | IHD | 1.598 | No harmful collinearity |
| Derivation cohort | Fully adjusted covariates | AKI | 1.590 | No harmful collinearity |
| Derivation cohort | Fully adjusted covariates | albumin | 1.531 | No harmful collinearity |
| Derivation cohort | Fully adjusted covariates | age | 1.517 | No harmful collinearity |
| Derivation cohort | Fully adjusted covariates | heart_failure | 1.465 | No harmful collinearity |
| Derivation cohort | Fully adjusted covariates | calcium_total | 1.446 | No harmful collinearity |
| Derivation cohort | Fully adjusted covariates | hypertension | 1.413 | No harmful collinearity |
| Derivation cohort | Fully adjusted covariates | MI | 1.367 | No harmful collinearity |
| Derivation cohort | Fully adjusted covariates | T2DM | 1.253 | No harmful collinearity |
| Derivation cohort | Fully adjusted covariates | anion_gap | 1.242 | No harmful collinearity |
| Derivation cohort | Fully adjusted covariates | gender_M | 1.172 | No harmful collinearity |
| Derivation cohort | Fully adjusted covariates | glucose | 1.168 | No harmful collinearity |
| Derivation cohort | Fully adjusted covariates | INR | 1.142 | No harmful collinearity |
| Derivation cohort | Fully adjusted covariates | potassium | 1.139 | No harmful collinearity |
| Derivation cohort | Fully adjusted covariates | bilirubin_total | 1.116 | No harmful collinearity |
| Derivation cohort | Fully adjusted covariates | pneumonia | 1.099 | No harmful collinearity |
| Derivation cohort | Fully adjusted covariates | COPD | 1.083 | No harmful collinearity |
| External validation cohort | Fully adjusted covariates | ALT | 3.455 | No harmful collinearity |
| External validation cohort | Fully adjusted covariates | AST | 3.349 | No harmful collinearity |
| External validation cohort | Fully adjusted covariates | sodium | 2.861 | No harmful collinearity |
| External validation cohort | Fully adjusted covariates | chloride | 2.822 | No harmful collinearity |
| External validation cohort | Fully adjusted covariates | urea_nitrogen | 2.176 | No harmful collinearity |
| External validation cohort | Fully adjusted covariates | creatinine | 2.158 | No harmful collinearity |
| External validation cohort | Fully adjusted covariates | IHD | 1.199 | No harmful collinearity |
| External validation cohort | Fully adjusted covariates | hypertension | 1.196 | No harmful collinearity |
| External validation cohort | Fully adjusted covariates | potassium | 1.170 | No harmful collinearity |
| External validation cohort | Fully adjusted covariates | INR | 1.156 | No harmful collinearity |
| External validation cohort | Fully adjusted covariates | CKD | 1.151 | No harmful collinearity |
| External validation cohort | Fully adjusted covariates | bilirubin_total | 1.118 | No harmful collinearity |
| External validation cohort | Fully adjusted covariates | heart_failure | 1.110 | No harmful collinearity |
| External validation cohort | Fully adjusted covariates | MI | 1.107 | No harmful collinearity |
| External validation cohort | Fully adjusted covariates | anion_gap | 1.103 | No harmful collinearity |
| External validation cohort | Fully adjusted covariates | T2DM | 1.086 | No harmful collinearity |
| External validation cohort | Fully adjusted covariates | albumin | 1.061 | No harmful collinearity |
| External validation cohort | Fully adjusted covariates | AKI | 1.058 | No harmful collinearity |
| External validation cohort | Fully adjusted covariates | gender_M | 1.036 | No harmful collinearity |
| External validation cohort | Fully adjusted covariates | glucose | 1.033 | No harmful collinearity |
| External validation cohort | Fully adjusted covariates | COPD | 1.032 | No harmful collinearity |
| External validation cohort | Fully adjusted covariates | pneumonia | 1.029 | No harmful collinearity |
| External validation cohort | Fully adjusted covariates | calcium_total | 1.027 | No harmful collinearity |
| External validation cohort | Fully adjusted covariates | age | 1.020 | No harmful collinearity |

VIF, variance inflation factor. The fully adjusted model included age, sex, race, acute kidney injury, chronic kidney disease, chronic obstructive pulmonary disease, hypertension, ischaemic heart disease, myocardial infarction, heart failure, pneumonia, type 2 diabetes mellitus, albumin, anion gap, creatinine, blood urea nitrogen, total bilirubin, glucose, sodium, potassium, chloride, total calcium, international normalised ratio, aspartate aminotransferase, and alanine aminotransferase. A VIF value below 5 was considered to indicate no harmful multicollinearity.
