# Supplementary material for: Comparative predictive value of nine inflammation-derived haematological indices for 28-day mortality in patients with sepsis: a multicentre retrospective cohort study
Source: Front Med (Lausanne). 2026 Jun 19;13:1857973. doi: 10.3389/fmed.2026.1857973 (PMC13328474; doi:10.3389/fmed.2026.1857973)
Supplement: Supplementary file 1 [file Data_Sheet_1.ZIP › Supplementary Files/Supplementary Table S3-2.docx]

**Supplementary Table S3-2. Variance Inflation Factor Summary After Adding Each Inflammation-Derived Haematological Index**

| **Cohort** | **Inflammation Index** | **VIF of Index** | **Maximum VIF in Model** | **Variable with Maximum VIF** | **Interpretation** |
| --- | --- | --- | --- | --- | --- |
| Derivation cohort | MP | 1.048 | 2.776 | race_WHITE | No harmful collinearity |
| Derivation cohort | MLR | 1.127 | 2.779 | race_WHITE | No harmful collinearity |
| Derivation cohort | SIRI | 1.140 | 2.778 | race_WHITE | No harmful collinearity |
| Derivation cohort | NLR | 1.119 | 2.779 | race_WHITE | No harmful collinearity |
| Derivation cohort | NM | 1.034 | 2.776 | race_WHITE | No harmful collinearity |
| Derivation cohort | AISI | 1.113 | 2.778 | race_WHITE | No harmful collinearity |
| Derivation cohort | NP | 1.079 | 2.777 | race_WHITE | No harmful collinearity |
| Derivation cohort | SII | 1.091 | 2.778 | race_WHITE | No harmful collinearity |
| Derivation cohort | PLR | 1.040 | 2.776 | race_WHITE | No harmful collinearity |
| External validation cohort | MP | 1.071 | 3.459 | ALT | No harmful collinearity |
| External validation cohort | MLR | 1.023 | 3.455 | ALT | No harmful collinearity |
| External validation cohort | SIRI | 1.032 | 3.465 | ALT | No harmful collinearity |
| External validation cohort | NLR | 1.049 | 3.461 | ALT | No harmful collinearity |
| External validation cohort | NM | 1.034 | 3.457 | ALT | No harmful collinearity |
| External validation cohort | AISI | 1.054 | 3.459 | ALT | No harmful collinearity |
| External validation cohort | NP | 1.062 | 3.508 | ALT | No harmful collinearity |
| External validation cohort | SII | 1.074 | 3.455 | ALT | No harmful collinearity |
| External validation cohort | PLR | 1.088 | 3.456 | ALT | No harmful collinearity |

Each inflammation-derived haematological index was standardised and added separately to the fully adjusted Cox model. The VIF of index indicates the variance inflation factor of the corresponding standardised inflammatory index. Maximum VIF in model refers to the highest VIF among all variables in that model. A VIF value below 5 was considered to indicate no harmful multicollinearity.
