# Supplementary material for: Comparative predictive value of nine inflammation-derived haematological indices for 28-day mortality in patients with sepsis: a multicentre retrospective cohort study
Source: Front Med (Lausanne). 2026 Jun 19;13:1857973. doi: 10.3389/fmed.2026.1857973 (PMC13328474; doi:10.3389/fmed.2026.1857973)
Supplement: Supplementary file 1 [file Data_Sheet_1.ZIP › Supplementary Files/Supplementary Table S4.docx]

**Supplementary Table S4. Stability of adjusted hazard ratios after excluding physiologically correlated covariates**

| **Cohort** | **Index** | **Full model HR (95% CI)** | **Reduced-collinearity model HR (95% CI)** | **Percent change in HR** | **P value in full model** | **P value in reduced model** | **Interpretation** |
| --- | --- | --- | --- | --- | --- | --- | --- |
| Derivation cohort | NLR | 1.221 (1.189-1.254) | 1.238 (1.205-1.271) | 1.4% | <0.001 | <0.001 | Stable |
| Derivation cohort | PLR | 1.141 (1.108-1.176) | 1.153 (1.119-1.187) | 1.0% | <0.001 | <0.001 | Stable |
| Derivation cohort | MLR | 1.232 (1.197-1.267) | 1.252 (1.217-1.288) | 1.7% | <0.001 | <0.001 | Stable |
| Derivation cohort | SII | 1.177 (1.145-1.209) | 1.189 (1.157-1.222) | 1.1% | <0.001 | <0.001 | Stable |
| Derivation cohort | SIRI | 1.212 (1.180-1.245) | 1.226 (1.194-1.259) | 1.2% | <0.001 | <0.001 | Stable |
| Derivation cohort | AISI | 1.167 (1.136-1.199) | 1.178 (1.147-1.210) | 0.9% | <0.001 | <0.001 | Stable |
| Derivation cohort | NM | 1.068 (1.033-1.104) | 1.064 (1.030-1.100) | 0.3% | <0.001 | <0.001 | Stable |
| Derivation cohort | NP | 1.223 (1.188-1.259) | 1.234 (1.199-1.270) | 0.8% | <0.001 | <0.001 | Stable |
| Derivation cohort | MP | 1.160 (1.128-1.193) | 1.172 (1.140-1.206) | 1.1% | <0.001 | <0.001 | Stable |

Note: The full model adjusted for age, sex, race, hypertension, acute kidney injury, pneumonia, cerebrovascular disease-related comorbidities where available, chronic kidney disease, diabetes, hyperlipidemia-related comorbidities where available, heart failure, myocardial infarction, ischemic heart disease, COPD, albumin, anion gap, creatinine, blood urea nitrogen, bilirubin, glucose, sodium, potassium, chloride, total calcium, INR, AST, and ALT, depending on available variables. The reduced-collinearity model removed chloride, blood urea nitrogen, and ALT while retaining sodium, creatinine, and AST. Inflammatory indices were winsorized at the 1st and 99th percentiles and entered as standardized continuous variables per 1-SD increase. Percent change was calculated as |HRreduced - HRfull| / HRfull × 100%; changes <10% were interpreted as stable.
