# Supplementary material for: Comparative predictive value of nine inflammation-derived haematological indices for 28-day mortality in patients with sepsis: a multicentre retrospective cohort study
Source: Front Med (Lausanne). 2026 Jun 19;13:1857973. doi: 10.3389/fmed.2026.1857973 (PMC13328474; doi:10.3389/fmed.2026.1857973)
Supplement: Supplementary file 1 [file Data_Sheet_1.ZIP › Supplementary Files/Supplementary Table S5.docx]

**Supplementary Table S5-1. Threshold ranges with net benefit over treat-all and treat-none (fully adjusted models)**

| **Model** | **Threshold range with net benefit over treat-all and treat-none** | **Interpretation** |
| --- | --- | --- |
| NLR | 1%-25% | Clinically useful |
| PLR | 1%-25% | Clinically useful |
| MLR | 1%-25% | Clinically useful |
| SII | 1%-25% | Clinically useful |
| SIRI | 1%-25% | Clinically useful |
| AISI | 1%-25% | Clinically useful |
| NM | 1%-25% | Clinically useful |
| NP | 1%-25% | Clinically useful |
| MP | 1%-25% | Clinically useful |

**Supplementary Table S5-2. Net benefit at clinically relevant threshold probabilities (fully adjusted models)**

| **Model** | **5%** | **10%** | **15%** | **20%** | **25%** | **Clinically useful range** |
| --- | --- | --- | --- | --- | --- | --- |
| Treat all | 0.0533 | 0.0007 | -0.0580 | -0.1242 | -0.1991 | - |
| Treat none | 0.0000 | 0.0000 | 0.0000 | 0.0000 | 0.0000 | - |
| NLR | 0.0712 | 0.0519 | 0.0393 | 0.0281 | 0.0201 | 1%-25% |
| PLR | 0.0701 | 0.0510 | 0.0379 | 0.0278 | 0.0183 | 1%-25% |
| MLR | 0.0709 | 0.0518 | 0.0390 | 0.0286 | 0.0196 | 1%-25% |
| SII | 0.0707 | 0.0510 | 0.0387 | 0.0280 | 0.0186 | 1%-25% |
| SIRI | 0.0710 | 0.0516 | 0.0394 | 0.0279 | 0.0198 | 1%-25% |
| AISI | 0.0707 | 0.0509 | 0.0386 | 0.0281 | 0.0188 | 1%-25% |
| NM | 0.0697 | 0.0507 | 0.0375 | 0.0271 | 0.0183 | 1%-25% |
| NP | 0.0700 | 0.0515 | 0.0379 | 0.0271 | 0.0195 | 1%-25% |
| MP | 0.0702 | 0.0512 | 0.0373 | 0.0268 | 0.0188 | 1%-25% |

**Supplementary Table S5-3. Threshold ranges with net benefit over treat-all and treat-none (all model specifications)**

| **Model specification** | **Model** | **Threshold range with net benefit over treat-all and treat-none** | **Interpretation** |
| --- | --- | --- | --- |
| Unadjusted | NLR | 8%-25% | Clinically useful |
| Unadjusted | PLR | 10%-24% | Clinically useful |
| Unadjusted | MLR | 7%-25% | Clinically useful |
| Unadjusted | SII | 8%-25% | Clinically useful |
| Unadjusted | SIRI | 8%-25% | Clinically useful |
| Unadjusted | AISI | 8%-25% | Clinically useful |
| Unadjusted | NM | 10%-14% | Clinically useful |
| Unadjusted | NP | 8%-25% | Clinically useful |
| Unadjusted | MP | 9%-25% | Clinically useful |
| Demographics | NLR | 2%-25% | Clinically useful |
| Demographics | PLR | 2%-25% | Clinically useful |
| Demographics | MLR | 2%-25% | Clinically useful |
| Demographics | SII | 2%-25% | Clinically useful |
| Demographics | SIRI | 2%-25% | Clinically useful |
| Demographics | AISI | 2%-25% | Clinically useful |
| Demographics | NM | 2%-25% | Clinically useful |
| Demographics | NP | 2%-25% | Clinically useful |
| Demographics | MP | 2%-25% | Clinically useful |
| Fully adjusted | NLR | 1%-25% | Clinically useful |
| Fully adjusted | PLR | 1%-25% | Clinically useful |
| Fully adjusted | MLR | 1%-25% | Clinically useful |
| Fully adjusted | SII | 1%-25% | Clinically useful |
| Fully adjusted | SIRI | 1%-25% | Clinically useful |
| Fully adjusted | AISI | 1%-25% | Clinically useful |
| Fully adjusted | NM | 1%-25% | Clinically useful |
| Fully adjusted | NP | 1%-25% | Clinically useful |
| Fully adjusted | MP | 1%-25% | Clinically useful |

**Supplementary Table S5-4. Net benefit at clinically relevant threshold probabilities (all model specifications)**

| **Model specification** | **Model** | **5%** | **10%** | **15%** | **20%** | **25%** | **Clinically useful range** |
| --- | --- | --- | --- | --- | --- | --- | --- |
| Reference | Treat all | 0.0533 | 0.0007 | -0.0580 | -0.1242 | -0.1991 | - |
| Reference | Treat none | 0.0000 | 0.0000 | 0.0000 | 0.0000 | 0.0000 | - |
| Unadjusted | NLR | 0.0533 | 0.0305 | 0.0105 | 0.0050 | 0.0023 | 8%-25% |
| Unadjusted | PLR | 0.0533 | 0.0170 | 0.0021 | 0.0005 | -0.0002 | 10%-24% |
| Unadjusted | MLR | 0.0533 | 0.0278 | 0.0095 | 0.0041 | 0.0023 | 7%-25% |
| Unadjusted | SII | 0.0533 | 0.0246 | 0.0077 | 0.0030 | 0.0016 | 8%-25% |
| Unadjusted | SIRI | 0.0533 | 0.0309 | 0.0117 | 0.0050 | 0.0024 | 8%-25% |
| Unadjusted | AISI | 0.0533 | 0.0235 | 0.0085 | 0.0038 | 0.0018 | 8%-25% |
| Unadjusted | NM | 0.0533 | 0.0073 | -0.0004 | -0.0002 | -0.0002 | 10%-14% |
| Unadjusted | NP | 0.0533 | 0.0222 | 0.0069 | 0.0031 | 0.0016 | 8%-25% |
| Unadjusted | MP | 0.0533 | 0.0161 | 0.0041 | 0.0018 | 0.0010 | 9%-25% |
| Demographics | NLR | 0.0590 | 0.0321 | 0.0195 | 0.0102 | 0.0051 | 2%-25% |
| Demographics | PLR | 0.0564 | 0.0256 | 0.0115 | 0.0048 | 0.0012 | 2%-25% |
| Demographics | MLR | 0.0589 | 0.0312 | 0.0183 | 0.0088 | 0.0038 | 2%-25% |
| Demographics | SII | 0.0570 | 0.0292 | 0.0166 | 0.0093 | 0.0041 | 2%-25% |
| Demographics | SIRI | 0.0590 | 0.0330 | 0.0196 | 0.0100 | 0.0046 | 2%-25% |
| Demographics | AISI | 0.0571 | 0.0293 | 0.0168 | 0.0090 | 0.0045 | 2%-25% |
| Demographics | NM | 0.0567 | 0.0235 | 0.0082 | 0.0026 | 0.0007 | 2%-25% |
| Demographics | NP | 0.0591 | 0.0293 | 0.0144 | 0.0069 | 0.0035 | 2%-25% |
| Demographics | MP | 0.0583 | 0.0262 | 0.0113 | 0.0047 | 0.0016 | 2%-25% |
| Fully adjusted | NLR | 0.0712 | 0.0519 | 0.0393 | 0.0281 | 0.0201 | 1%-25% |
| Fully adjusted | PLR | 0.0701 | 0.0510 | 0.0379 | 0.0278 | 0.0183 | 1%-25% |
| Fully adjusted | MLR | 0.0709 | 0.0518 | 0.0390 | 0.0286 | 0.0196 | 1%-25% |
| Fully adjusted | SII | 0.0707 | 0.0510 | 0.0387 | 0.0280 | 0.0186 | 1%-25% |
| Fully adjusted | SIRI | 0.0710 | 0.0516 | 0.0394 | 0.0279 | 0.0198 | 1%-25% |
| Fully adjusted | AISI | 0.0707 | 0.0509 | 0.0386 | 0.0281 | 0.0188 | 1%-25% |
| Fully adjusted | NM | 0.0697 | 0.0507 | 0.0375 | 0.0271 | 0.0183 | 1%-25% |
| Fully adjusted | NP | 0.0700 | 0.0515 | 0.0379 | 0.0271 | 0.0195 | 1%-25% |
| Fully adjusted | MP | 0.0702 | 0.0512 | 0.0373 | 0.0268 | 0.0188 | 1%-25% |

Note: Net benefit was summarized from the decision curve analysis for 28-day mortality after the 24-h landmark. The observed event prevalence was 0.1007. A model was considered clinically useful at a given threshold probability when its net benefit was greater than both treat-all and treat-none. Clinically useful ranges were assessed within threshold probabilities from 1% to 25%.
