# Supplementary material for: Comparative predictive value of nine inflammation-derived haematological indices for 28-day mortality in patients with sepsis: a multicentre retrospective cohort study
Source: Front Med (Lausanne). 2026 Jun 19;13:1857973. doi: 10.3389/fmed.2026.1857973 (PMC13328474; doi:10.3389/fmed.2026.1857973)
Supplement: Supplementary file 1 [file Data_Sheet_1.ZIP › Supplementary Files/Supplementary Table S6.docx]

**Supplementary Table S6. Sensitivity analysis comparing imputed and complete-case datasets**

| **Index** | **Imputed dataset HR (95% CI)** | **Complete-case dataset HR (95% CI)** | **Percent change in HR** | **P value in imputed dataset** | **P value in complete-case dataset** | **Interpretation** |
| --- | --- | --- | --- | --- | --- | --- |
| NLR | 1.221 (1.189-1.254) | 1.217 (1.187-1.249) | 0.3% | <0.001 | <0.001 | Stable |
| PLR | 1.142 (1.109-1.176) | 1.142 (1.108-1.176) | 0.0% | <0.001 | <0.001 | Stable |
| MLR | 1.232 (1.197-1.267) | 1.231 (1.198-1.265) | 0.0% | <0.001 | <0.001 | Stable |
| SII | 1.177 (1.145-1.210) | 1.164 (1.134-1.195) | 1.1% | <0.001 | <0.001 | Stable |
| SIRI | 1.213 (1.181-1.245) | 1.184 (1.155-1.213) | 2.4% | <0.001 | <0.001 | Stable |
| AISI | 1.168 (1.137-1.200) | 1.139 (1.111-1.168) | 2.5% | <0.001 | <0.001 | Stable |
| NM | 1.068 (1.033-1.103) | 1.077 (1.044-1.111) | 0.9% | <0.001 | <0.001 | Stable |
| NP | 1.223 (1.188-1.258) | 1.204 (1.171-1.237) | 1.5% | <0.001 | <0.001 | Stable |
| MP | 1.159 (1.127-1.192) | 1.151 (1.121-1.182) | 0.7% | <0.001 | <0.001 | Stable |

Note: Landmark outcomes were regenerated using the same 24-h landmark rule. Patients who died or were discharged before 24 h after admission were excluded; follow-up started at the 24-h landmark and was administratively censored at 28 days. Inflammatory indices were winsorized at the 1st and 99th percentiles and entered as standardized continuous variables per 1-SD increase. The same fully adjusted Cox model was fitted in the imputed and complete-case datasets.
