# Supplementary material for: Comparative predictive value of nine inflammation-derived haematological indices for 28-day mortality in patients with sepsis: a multicentre retrospective cohort study
Source: Front Med (Lausanne). 2026 Jun 19;13:1857973. doi: 10.3389/fmed.2026.1857973 (PMC13328474; doi:10.3389/fmed.2026.1857973)
Supplement: Supplementary file 1 [file Data_Sheet_1.ZIP › Supplementary Files/Supplementary Table S7.docx]

**Supplementary Table S7. Proportional hazards assumption testing for the fully adjusted Cox models**

| **Index** | **Global Schoenfeld test P value** | **Index-specific Schoenfeld test P value** | **PH assumption** |
| --- | --- | --- | --- |
| NLR | <0.001 | 0.703 | Global test significant; index-specific PH not violated |
| PLR | <0.001 | 0.798 | Global test significant; index-specific PH not violated |
| MLR | <0.001 | 0.853 | Global test significant; index-specific PH not violated |
| SII | <0.001 | 0.344 | Global test significant; index-specific PH not violated |
| SIRI | <0.001 | 0.305 | Global test significant; index-specific PH not violated |
| AISI | <0.001 | 0.129 | Global test significant; index-specific PH not violated |
| NM | <0.001 | 0.535 | Global test significant; index-specific PH not violated |
| NP | <0.001 | 0.111 | Global test significant; index-specific PH not violated |
| MP | <0.001 | 0.656 | Global test significant; index-specific PH not violated |

Note: The proportional hazards assumption was assessed using Schoenfeld residuals for each fully adjusted Cox model. Inflammatory indices were winsorized at the 1st and 99th percentiles and entered as standardized continuous variables per 1-SD increase. A two-sided P value > 0.05 was considered to indicate no evidence of violation of the proportional hazards assumption. The global test evaluates all covariates in the model jointly, whereas the index-specific test evaluates the inflammatory index term only.
