# Supplementary material for: Comparative predictive value of nine inflammation-derived haematological indices for 28-day mortality in patients with sepsis: a multicentre retrospective cohort study
Source: Front Med (Lausanne). 2026 Jun 19;13:1857973. doi: 10.3389/fmed.2026.1857973 (PMC13328474; doi:10.3389/fmed.2026.1857973)
Supplement: Supplementary file 1 [file Data_Sheet_1.ZIP › Supplementary Files/Supplementary Table S8.docx]

**Supplementary Table S8. Sensitivity analysis of winsorisation for inflammatory markers**

| **Index** | **Non-winsorized HR (95% CI)** | **Winsorized HR (95% CI)** | **Percent change in HR** | **P value without winsorisation** | **P value with winsorisation** | **Interpretation** |
| --- | --- | --- | --- | --- | --- | --- |
| NLR | 1.082 (1.071-1.093) | 1.221 (1.189-1.254) | 12.8% | <0.001 | <0.001 | Changed |
| PLR | 1.034 (1.022-1.046) | 1.141 (1.108-1.176) | 10.4% | <0.001 | <0.001 | Changed |
| MLR | 1.135 (1.112-1.158) | 1.232 (1.197-1.267) | 8.5% | <0.001 | <0.001 | Stable |
| SII | 1.059 (1.049-1.070) | 1.177 (1.145-1.209) | 11.1% | <0.001 | <0.001 | Changed |
| SIRI | 1.133 (1.110-1.156) | 1.212 (1.180-1.245) | 7.0% | <0.001 | <0.001 | Stable |
| AISI | 1.110 (1.087-1.133) | 1.167 (1.136-1.199) | 5.2% | <0.001 | <0.001 | Stable |
| NM | 1.053 (1.021-1.085) | 1.068 (1.033-1.104) | 1.4% | <0.001 | <0.001 | Stable |
| NP | 1.081 (1.065-1.097) | 1.223 (1.188-1.259) | 13.2% | <0.001 | <0.001 | Changed |
| MP | 1.080 (1.065-1.096) | 1.160 (1.128-1.193) | 7.3% | <0.001 | <0.001 | Stable |
