# Supplementary material for: Comparative predictive value of nine inflammation-derived haematological indices for 28-day mortality in patients with sepsis: a multicentre retrospective cohort study
Source: Front Med (Lausanne). 2026 Jun 19;13:1857973. doi: 10.3389/fmed.2026.1857973 (PMC13328474; doi:10.3389/fmed.2026.1857973)
Supplement: Supplementary file 1 [file Data_Sheet_1.ZIP › Supplementary Files/Supplementary Table S9.docx]

**Supplementary Table S9-1. Discrimination improvement of SOFA plus inflammatory markers.**

| **Model** | **AUC (95% CI)** | **Delta AUC vs SOFA** | **P for Delta AUC** |
| --- | --- | --- | --- |
| SOFA alone | 0.582 (0.570-0.594) | Reference | - |
| SOFA + NLR | 0.705 (0.693-0.716) | 0.123 | <0.001 |
| SOFA + PLR | 0.637 (0.625-0.648) | 0.055 | <0.001 |
| SOFA + MLR | 0.691 (0.681-0.702) | 0.109 | <0.001 |
| SOFA + SII | 0.678 (0.668-0.689) | 0.096 | <0.001 |
| SOFA + SIRI | 0.698 (0.687-0.709) | 0.116 | <0.001 |
| SOFA + AISI | 0.673 (0.662-0.685) | 0.091 | <0.001 |
| SOFA + NM | 0.594 (0.582-0.605) | 0.012 | <0.001 |
| SOFA + NP | 0.657 (0.646-0.669) | 0.075 | <0.001 |
| SOFA + MP | 0.621 (0.609-0.632) | 0.039 | <0.001 |

**Supplementary Table S9-2. Likelihood ratio tests for nested Cox models.**

| **Model comparison** | **Chi-square** | **df** | **P value** |
| --- | --- | --- | --- |
| SOFA alone vs SOFA + NLR | 939.565 | 1 | <0.001 |
| SOFA alone vs SOFA + PLR | 303.904 | 1 | <0.001 |
| SOFA alone vs SOFA + MLR | 842.106 | 1 | <0.001 |
| SOFA alone vs SOFA + SII | 687.983 | 1 | <0.001 |
| SOFA alone vs SOFA + SIRI | 940.240 | 1 | <0.001 |
| SOFA alone vs SOFA + AISI | 670.218 | 1 | <0.001 |
| SOFA alone vs SOFA + NM | 64.307 | 1 | <0.001 |
| SOFA alone vs SOFA + NP | 667.185 | 1 | <0.001 |
| SOFA alone vs SOFA + MP | 364.939 | 1 | <0.001 |

**Supplementary Table S9-3. IDI and continuous NRI for SOFA plus inflammatory markers.**

| **Model** | **IDI (95% CI)** | **P for IDI** | **continuous NRI (95% CI)** | **P for NRI** |
| --- | --- | --- | --- | --- |
| SOFA + NLR | 0.044 (0.040-0.048) | <0.001 | 0.575 (0.537-0.612) | <0.001 |
| SOFA + PLR | 0.013 (0.011-0.015) | <0.001 | 0.338 (0.300-0.378) | <0.001 |
| SOFA + MLR | 0.039 (0.036-0.043) | <0.001 | 0.532 (0.491-0.571) | <0.001 |
| SOFA + SII | 0.031 (0.028-0.035) | <0.001 | 0.484 (0.446-0.523) | <0.001 |
| SOFA + SIRI | 0.045 (0.041-0.049) | <0.001 | 0.586 (0.548-0.626) | <0.001 |
| SOFA + AISI | 0.031 (0.028-0.035) | <0.001 | 0.462 (0.422-0.499) | <0.001 |
| SOFA + NM | 0.003 (0.002-0.004) | <0.001 | 0.120 (0.084-0.158) | <0.001 |
| SOFA + NP | 0.035 (0.031-0.039) | <0.001 | 0.392 (0.352-0.429) | <0.001 |
| SOFA + MP | 0.020 (0.017-0.023) | <0.001 | 0.287 (0.250-0.328) | <0.001 |

Abbreviations: AUC, area under the curve; CI, confidence interval; IDI, integrated discrimination improvement; NRI, net reclassification improvement; SOFA, Sequential Organ Failure Assessment. Inflammatory markers were winsorized at the 1st and 99th percentiles and entered as standardized continuous variables per 1-SD increase.
